# Functional convergence in Z-containing DNA biosynthesis highlighted by the characterization of nucleotide metabolism enzymes in bacteriophages

**DOI:** 10.1093/nar/gkag079

**Published:** 2026-02-09

**Authors:** Florent Poubanne, Ekaterina Darii, Aline Mariage, Eddy Elisée, Peggy Sirvain, Camille Hassan, Julie Rivollier, Aurélie Fossey-Jouenne, Alain Perret, Raphaël Méheust, Valérie Pezo

**Affiliations:** Génomique Métabolique, Genoscope, Institut François Jacob, CEA, CNRS, Univ Evry, Université Paris-Saclay, 2 rue Gaston Crémieux, Evry 91057, France; Génomique Métabolique, Genoscope, Institut François Jacob, CEA, CNRS, Univ Evry, Université Paris-Saclay, 2 rue Gaston Crémieux, Evry 91057, France; Génomique Métabolique, Genoscope, Institut François Jacob, CEA, CNRS, Univ Evry, Université Paris-Saclay, 2 rue Gaston Crémieux, Evry 91057, France; Génomique Métabolique, Genoscope, Institut François Jacob, CEA, CNRS, Univ Evry, Université Paris-Saclay, 2 rue Gaston Crémieux, Evry 91057, France; Génomique Métabolique, Genoscope, Institut François Jacob, CEA, CNRS, Univ Evry, Université Paris-Saclay, 2 rue Gaston Crémieux, Evry 91057, France; Génomique Métabolique, Genoscope, Institut François Jacob, CEA, CNRS, Univ Evry, Université Paris-Saclay, 2 rue Gaston Crémieux, Evry 91057, France; Génomique Métabolique, Genoscope, Institut François Jacob, CEA, CNRS, Univ Evry, Université Paris-Saclay, 2 rue Gaston Crémieux, Evry 91057, France; TESSSI, 81 rue Réaumur, Paris 75002, France; Génomique Métabolique, Genoscope, Institut François Jacob, CEA, CNRS, Univ Evry, Université Paris-Saclay, 2 rue Gaston Crémieux, Evry 91057, France; Génomique Métabolique, Genoscope, Institut François Jacob, CEA, CNRS, Univ Evry, Université Paris-Saclay, 2 rue Gaston Crémieux, Evry 91057, France; Génomique Métabolique, Genoscope, Institut François Jacob, CEA, CNRS, Univ Evry, Université Paris-Saclay, 2 rue Gaston Crémieux, Evry 91057, France; Génomique Métabolique, Genoscope, Institut François Jacob, CEA, CNRS, Univ Evry, Université Paris-Saclay, 2 rue Gaston Crémieux, Evry 91057, France

## Abstract

Certain DNA bacteriophages exhibit a complete substitution of their genomic adenine (A) by 2-aminoadenine (Z), forming three hydrogen bonds with thymine. dZTP biosynthesis is performed by a phage-encoded 2-amino adenylosuccinate synthetase (PurZ) whereas a Z-specific DNA polymerase I (DpoZ) has been shown to incorporate the dZTP. Our investigations into the nucleotide metabolism of Z-bacteriophages, integrating modeling, biochemical, and phylogenetic approaches, reveal novel enzymatic activities. We characterized two distinct enzymes that both hydrolyze dATP and dGTP, and a DmtZ enzyme with dual activity. DmtZ acts as a dAMP-specific hydrolase, converting dAMP to adenine, and uniquely transfers deoxyribose 5-phosphate from dAMP to the Z base to produce dZMP, which is subsequently converted to dZTP. This dual functionality marks DmtZ as the first enzyme in the nucleoside deoxyribosyltransferase (NDT) family with such a mechanism and uncovers a novel biosynthetic route for dZTP. Phylogenetic analyses indicate multiple independent acquisitions of enzymes involved in nucleotide metabolism, occurring after PurZ acquisition, yet converging on equivalent metabolic functions. Deciphering these propagation mechanisms in DNA-modified bacteriophages illuminates functional diversity in viral metabolism and a striking example of functional convergence.

## Graphical Abstract

**Graphical Abstract:**
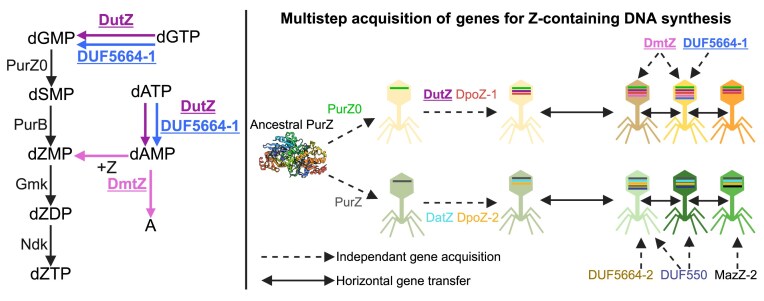

## Introduction

In the natural world, bacteriophage genomes display the greatest chemical diversity of noncanonical nucleobases [1, 2]. These modifications have been found for every DNA base and can result either from direct incorporation of the modified nucleotide during replication, or from modification of the nucleotide by specific enzymes after replication. Both mechanisms of DNA modification can involve the complete or partial replacement of a canonical base by a modified one. Enzymes encoded by bacteriophages can play a key role in enabling the synthesis of these modified genomes, by modulating the intracellular composition of canonical nucleotides or even inhibiting the function of certain cellular enzymes.

Some DNA viruses infecting hosts such as *Pseudomonadota, Cyanobacteriota*, and *Actinomycetota*, show a complete substitution of adenine (A) by 2-aminoadenine (Z) (Supplementary Fig. S1), thus forming three hydrogen bonds in Z:T pairs and deviating from the Watson-Crick rules. This substitution increases the DNA thermal stability compared with canonical DNA, and partially prevents cleavage by restriction endonucleases from the bacterial host [3]. The 2-amino deoxyadenosine triphosphate (dZTP) biosynthetic pathway has been elucidated for three bacteriophages containing ZTGC genomes: *Vibrio* phage phiVC8, *Acinetobacter* phage SH-Ab 15 497, and *Synechococcus* phage S-2L [4–6]. These phages encode a new PurZ enzyme, a distant paralog of adenylosuccinate synthetase (PurA). PurA catalyzes the first committed step towards adenine biosynthesis by condensing aspartate and inosine monophosphate (IMP) in the presence of guanosine triphosphate (GTP). PurZ condenses aspartate with deoxyguanylate (dGMP) in presence of adenosine triphosphate (ATP) into N6-succino-2-amino-2′-deoxyadenylate (dSMP) (Supplementary Fig. S1). Conversion of dSMP to dZMP, and then dZMP to the final dZTP is carried out by the bacterial host enzymes. In most of these bacteriophages harboring a PurZ, we identified and characterized a specific type I DNA polymerase (DpoZ) [7]. This DpoZ enzyme was therefore suggested to be responsible for the synthesis of Z-genomes. Alongside with the PurZ and DpoZ, these bacteriophages harbored numerous proteins likely to be involved in nucleotide metabolism. Studies carried out on the *Acinetobacter* SH-Ab 15 497 and *Synechococcus* S-2L phages showed that specific enzymes are specialized in nucleotide pool modification to favor Z-containing DNA synthesis [4, 6, 8].

In the arms race between host and phage, the latter requires to develop unique properties or mechanisms of action, selected by the need to propagate its DNA. This feature makes phage proteins an interesting target for novel functions. New enzymatic activities involved in nucleotide metabolism are of major interest for many biotechnological applications and particularly for the propagation of genetic information based on the use of non-natural nucleic acids (XNA for Xenobiotic Nucleic Acids) [9–11].

In the objective of discovering new enzymatic activities while trying to understand the mechanisms of bacteriophage propagation, we have combined modeling, biochemical and phylogenetic approaches to explore the enzymatic potential of these Z bacteriophages. Our study identified three genes in Z-genomes harboring a *purZ0* variant [12]. Biochemical characterization of these proteins revealed that two of these enzymes convert dATP and dGTP into dAMP and dGMP respectively to promote Z-containing DNA biosynthesis. The third enzyme, designated DmtZ, exhibits a unique dual activity: hydrolyzing dAMP to adenine and transferring deoxyribose phosphate of dAMP to a Z nucleobase thereby generating dZMP. In addition to revealing a new unsuspected pathway for dZTP biosynthesis, this enzyme with the capacity to transfer a deoxyribose phosphate on a nucleobase confirms the potential of bacteriophages to discover new functions. Evolutionary analyses revealed a single origin for PurZ but distinct evolutionary paths for nucleotide pool-regulating enzymes and DNA polymerase, suggesting functional convergence.

## Materials and methods

### Chemicals, primers, and culture medium

MgCl_2_, NaCl, glycerol, Trizma base, HCl, potassium carbonate, potassium bicarbonate, and HEPES were purchased from Sigma Aldrich, Dithiothreitol (DTT) from Biosolve, and nucleotides from Invitrogen and Trilink biotechnologies. Primers were synthesized by Eurofins Genomics. Bacteria were routinely grown in Luria–Bertani medium at 37°C with 30 mg/l of kanamycin when necessary.

### Proteins structure prediction and TM-calculation

The protein structures were predicted using Alphafold2 [13] and the ColabFold tool v1.5.2 [14]. TM-scores were calculated with TM-align [15] (update 15-04-2022).

### Cloning, production and purification of proteins

DutZ (AOE44406.1) and DmtZ (AOE44404.1) from *Gordonia* phage Ghobes as well as DmtZ-DutZ (ALY10777.1) and DUF5664 (ALY10775.1) from *Arthrobacter* phage Wayne were amplified by polymerase chain reaction from genomic DNA using primers couples X1888/X1889, X1890/X1891, X1892/X1893, and X3825/X4086, respectively (Supplementary Table 1). Amplicons were then digested with PacI or NdeI and NotI restriction enzymes and ligated with a PvuI-NotI-digested pGEN452 vector [7] or a NdeI-NotI-digested pET-24a (Novagen). These plasmids allow the addition of a 6-histidines tag at proteins N- or C-terminus, respectively. The N-ter and C-ter domains of DmtZ–DutZ from Arthrobacter phage Wayne were subcloned in pGEN452 vector from the precedently depicted plasmid coding for the whole protein with the primer couples X1892/X3442 and X3441/X1893 respectively. DmtZ Ghobes mutants E103A, P57A, N77D, Y6A, D12A, and D12R were made using primers couples X4151/X4152, X4167/X4168, X4031/X4032, X4371, X4149, and X4150, respectively, for site-directed mutagenesis (Supplementary Table 1). These plasmids were used to transform the C43 *Escherichia coli* strain (Sigma–Aldrich) by electroporation for protein production. Transformed cells were grown in 1 l of Terrific Broth medium containing 0.5 M sorbitol, 5 mM betaine, and 30 mg/l kanamycin at 37°C until reaching an *A*600 of 2. Isopropyl B-D-thiogalactopyranoside was added at a concentration of 500 µM to induce protein production, and the cells were further grown at 20°C overnight. After centrifugation, the cells were washed and suspended in 64 ml of lysis buffer (50 mM phosphate, pH 8.0, 500 mM NaCl, 15% glycerol, and 30 mM imidazole) containing 1 mM Pefabloc SC, 52 µl of LysonaseTM bioprocessing reagent (Novagen), and 3.8 ml of BugBuster (Millipore). After centrifugation, the supernatant was loaded onto a 15-ml HisTrap FF column (Cytivia, ref: 17525501) in the first purification step. The column was washed with the lysis buffer and the protein was eluted with the same buffer containing 250 mM imidazole. The eluted peak was redirected on a HiLoad 16/600 Superdex 200-pg size exclusion column (Cytivia, ref: 28989335) and collected in Tris–HCl, pH 7.5, 50 mM, NaCl 50 mM, DTT 1 mM, glycerol 15%.

### Enzymatic assays for liquid chromatography-high resolution mass spectrometry and UltraViolet - High Performance Liquid Chromatography (UV-HPLC) analysis

Hydrolase and dUTPase reactions were performed in a 20 µl mix containing Tris–HCl, pH 7.5, 25 mM, MgCl_2_ 5 mM, DTT 1 mM, nucleotide 1 mM, and 4 µM of enzyme at 37°C for 30 min. For transferase reactions, donor dAMP or dATP was at 3 mM and acceptor base at 1 mM with 4 µM of enzyme at 37°C for 1h in 20 µl mix containing Tris–HCl, pH 7.5, 25 mM, MgCl_2_ 5 mM, DTT 1 mM. DUF5664 reactions were performed in a 20 µl mix containing HEPES pH7.5 25 mM, MnCl_2_ 2 mM, dNTP 1 mM and 4 µM of enzymes at 37°C for 1h. All the reactions were stopped with 1 µl of trifluoroacetic acid 20%. pH was then neutralized with 2 µl of K_2_CO_3_ 1.56 M. Twenty-three microliter solutions were then diluted in 46 µl of a mix of 80% acetonitrile and 20% 10 mM ammonium carbonate buffer (pH 10) and filtered with 0.2 µm wwPTFE membrane plate filters from Cytiva. Hydrolase and dUTPase reactions were analysed by liquid chromatography-high resolution mass spectrometry (LC-HRMS). Transferase and DUF5664 reactions were analysed by Ultra high performance liquid chromatography UV (UHPLC-UV).

### LC-HRMS analysis

LC-HRMS analyses were performed using a Dionex™ Ultimate 3000 RS UHPLC system (Thermo Fisher Scientific) coupled to an Orbitrap Elite hybrid mass spectrometer (Thermo Fisher Scientific), operated in negative ionization mode with a heated electrospray ionization (HESI) source. Chromatographic separation was carried out on a Atlantis Premier BEH Z-HILIC column (1.7 µm, 2.1 × 150 mm; Waters™, Cat. No. 186009980), maintained at 40°C, with a constant flow rate of 0.5 ml/min. Chromatographic separation was performed using a binary mobile phase composed of acetonitrile and 10 mM ammonium carbonate buffer (pH 10). A 20 min linear gradient from 80:20 to 40:60 (v/v) acetonitrile:buffer was applied, followed by 7 min of isocratic elution at 40:60, a return to 80:20 ratio over 5 min, and 14 min of re-equilibration at 80:20.

A mass spectrometer equipped with a HESI source was operated with a spray voltage of −4.0 kV, sheath gas and auxiliary gas flow rates set to 55 and 40 arbitrary units (a.u.), respectively. The capillary and heater temperatures were both set to 275°C. Full-scan mass spectra were acquired over an m/z range of 50–1000 with a resolution of 60 000 (full width at half maximum at m/z 400). Data acquisition and processing were performed using the Qual Browser module of Xcalibur software version 2.2 (Thermo Fisher Scientific).

### UHPLC-UV analysis

UHPLC-UV analyses were carried out using a Dionex™ Ultimate 3000 RS UHPLC system (Thermo Fisher Scientific) equipped with a multi-wavelength ultraviolet (UV) detector. A volume of 3 µl of each sample (reaction mixtures or standards) was injected onto an Atlantis Premier BEH Z-HILIC column (1.7 µm, 2.1 × 150 mm; Waters™, Cat. No. 186009980), maintained at 30°C. Chromatographic separation was performed using a binary mobile phase composed of acetonitrile and 10 mM ammonium bicarbonate buffer (pH 10). A 14 min linear gradient from 80:20 to 40:60 (v/v) acetonitrile:buffer was applied, followed by 3 min of isocratic elution at 40:60, a return to 80:20 ratio over 2 min, and 10 min of re-equilibration at 80:20 for 14 min. The flow rate was set to 0.2 ml/min and UV absorbance was monitored at 260 nm.

### Kinetic characterization of DmtZ hydrolase activity using a spectrophotometric enzymatic assay

The hydrolase activity of DmtZ from Ghobes (wild-type and mutants) on dNMP substrates, producing 2-deoxyribose 5-phosphate, was determined by monitoring NADH oxidation at 340 nm using a coupled enzymatic assay, as previously described [16]. The reaction mixture contained 50 mM Tris–acetate pH 6, 5 mM MgCl_2_, 1 mM DTT, 0.2 mM NADH, 4.5 µM of purified aldolase from *E. coli* (DeoC), 1 unit of coupled enzymes triose-phosphate isomerase and glycerol-3-phosphate dehydrogenase (TPI-GPDH from rabbit muscle, Sigma–Aldrich), varying dNMP concentrations in a total volume of 100 µl. The reaction was initiated by the addition of the purified enzymes (DmtZ or mutants) and the decrease in absorbance at 340 nm was measured at 30°C in a SAFAS UVmc2 double-beam spectrophotometer combined with a heating circulator. All kinetic parameters were determined from triplicate experiments by nonlinear analysis of initial rates with SigmaPlot version 9.0 (Systat Software).

### Kinetic characterization of dNTP-pyrophosphohydrolase activity using a colorimetric enzymatic assay

The dNTP-pyrophosphohydrolase activity of DutZ and DUF5664 was quantified by measuring the release of phosphate from dNTP substrates. This measurement utilized an established inorganic pyrophosphatase-phosphomolybdate-malachite green assay [17], which monitors pyrophosphate production. Assays were conducted in 80 μl reaction mixtures containing 5 mM MgCl2, 0.3 μM yeast inorganic pyrophosphatase (Sigma–Aldrich), and various dNTP concentrations. The reaction buffer was 25 mM Tris–HCl (pH 7.5) for DutZ and 25 mM HEPES (pH 7.5) for DUF5664. Reactions were initiated by adding the purified enzyme at room temperature, quenched at specified time points (0, 2, 4, 6, 8, and 10 min) with 20 μl of the phosphomolybdate-malachite green reagent, and the resulting phosphate release was quantified by measuring absorbance at 630 nm using a SpectraMax Plus microplate reader. All kinetic parameters were determined from triplicate experiments using SigmaPlot version 14.5 (Systat Software).

### Molecular docking

The Ghobes DmtZ template used for the docking experiment was generated by Alphafold2 [13] and the ColabFold tool v1.5.2 [14]. The X-ray crystal structure of the deoxynucleoside 5-monophosphate *N*-glycosidase of *Rattus norvegicus* DNPH1 (PDB ID:4FYH) served as reference. The PyMOL Molecular Graphics System, Version 2.5.5 Schrödinger, LLC was used to align predicted apo-enzyme against reference holo-enzyme structure. The ligand coordinates were taken from the holo-enzyme structure and transferred to the predicted enzyme. The lowest energy model of predicted protein was then calculated using the YASARA Structure software [18, 19] (v 22.9.24). Original ligand was replaced by ligand of interest using the AutoDock Vina programme [20] from the DockingPie plugin (v1.2.1) [21]. The final lowest energy model was calculated again using YASARA.

### Phylogenetic trees construction

PurA proteins were retrieved using the Pfam accession PF00709 [22] and the hmmsearch tool [23] (v3.3.2) against the protein sequences from all NCBI viruses (downloaded on 12 June 2022) [24]. The PurZ structure of *Synechococcus* phage S2-L (PDB ID:7ODX) was aligned using the TM-align method hosted in the Protein DataBase (PDB) webserver [25] against the structures of PurZ of *Vibrio* phage phiVC8 (PDB ID:6FM1), PurZ0 of *Gordonia* phage Archimedes (PDB ID:7VF6), PurA of *Pyrococcus horikoshii* OT3 (PDB ID:5K7X) and PurA from *E. coli* K-12 (PDB ID:1CG0). The four pairwise alignments were then used to guide the alignment of the PurA sequences using the MAFFT webserver (version 7) [26]. The alignment was further trimmed using Trimal (v1.4.1) (–gappyout option) [27]. A phylogenetic tree was constructed with iqtree v1.6.12 [28] using ModelFinder [29] to select the best model of evolution, and with 1000 ultrafast bootstrap [30]. The phylogenetic tree was visualized with iTOL [31]. A similar protocol without structure guidance was used to build phylogenetic trees for NDT-like (hand-made HMM, see supplementary data), Yfbr-like (DatZ) (PFAM: PF12917.11), and DUF550 (PFAM: PF04447.15) proteins except that identical sequences were removed using CD-HIT [32] (v4.8.1). For Dut-like (PFAM: PF00692.22), DUF5664 (PFAM: PF18909.3), and DNA Polymerase I (PFAM: PF00476.24) trees, clusterings were performed at 90% of identity except for proteins encoded by PurA-homologs-possessing viruses for which no clustering has been applied.

### Nucleotides metabolism proteins presence/absence

Proteins encoded by Z-genomes were annotated based on the accession of their best Hmmsearch match against the Pfam database [22] (v37.1). Due to the lack of Pfam HMMs, two hand-made HMMs were built using hmmbuild for NDT-like and MazZ-2 proteins.

### Network-based phylogeny

The network of Z-genomes was constructed based on gene contents using vConTACT v.2.0 [33] using the following settings : “db ‘ProkaryoticViralRefSeq211-Merged’ –c1-bin cluster_one-1.0.jar –threads 36″.

## Results

### Identification of proteins involved in nucleotide metabolism in Gordonia phage Ghobes and Arthrobacter phage Wayne

**Figure 1.**
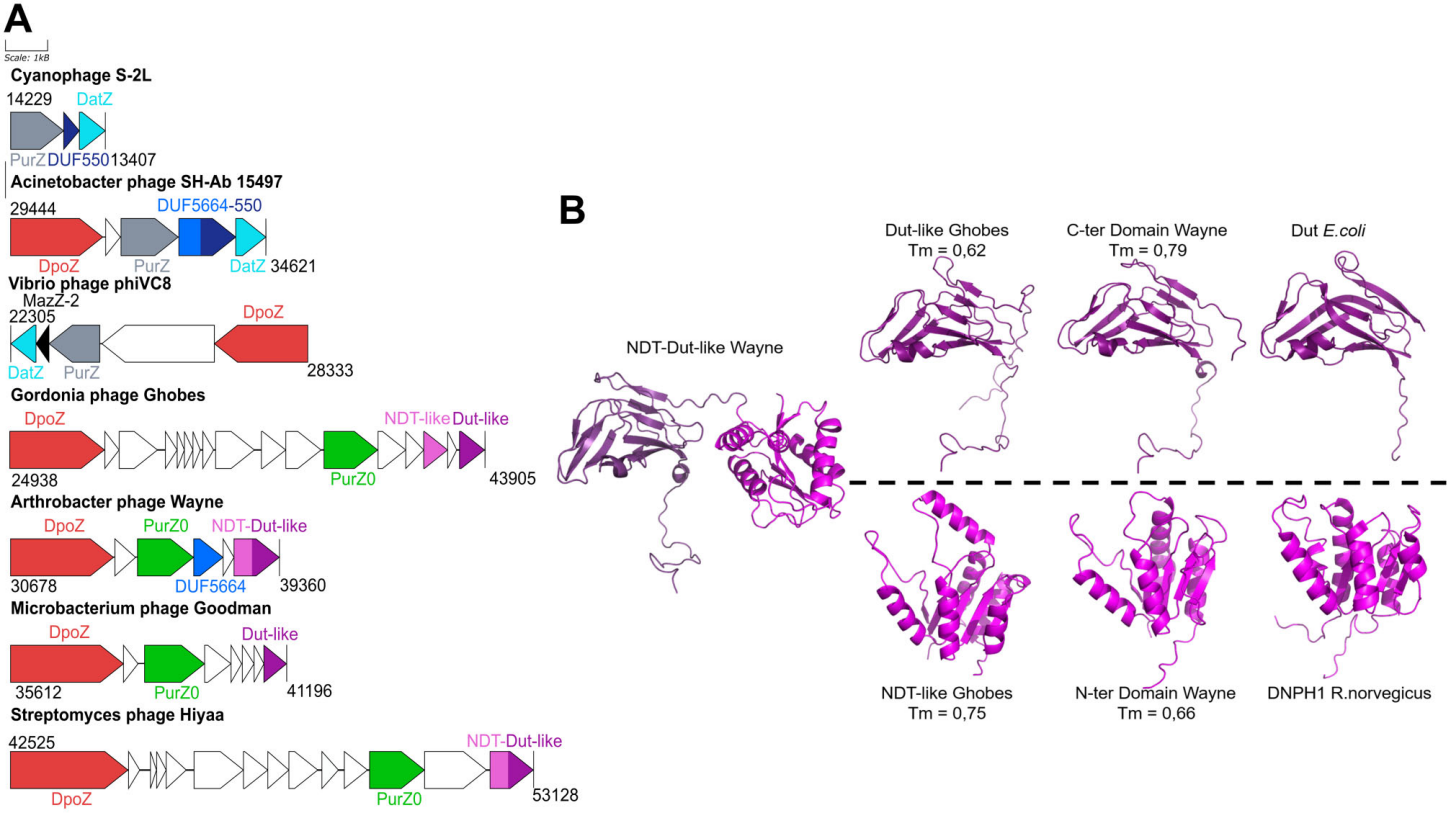
Genomic organisation of phages with Z-containing DNA and predicted structures of Dut-like and NDT-like proteins of PurZ0-containing phages. **(A)** Gene clusters involved in Z-containing DNA synthesis in representative phages containing PurZ (in grey) or PurZ0 (in green). **(B)** Comparison of Dut-like (pLDDT = 84.8; pTM = 0.746), NDT-like Ghobes (pLDDT = 83.5; pTM = 0.827) and NDT-Dut-like Wayne (pLDDT = 81.9; pTM = 0.498) domains predicted structure with their closest structural homologs Dut *E. coli* (PDB ID:1DUP) and DNPH1 *R. norvegicus* (PDB ID:2KHZ). pLDDT (predicted Local Distance Difference Test) reports residue-level confidence (values > 90: very high accuracy; 70–90: moderate to high confidence; <70: lower reliability). pTM (predicted Template Modeling score) reflects the expected accuracy of the global domain arrangement (values > 0.7: reliable overall fold; <0.7: increased uncertainty in domain positioning). Structural similarity scores (TM-scores) quantify the similarity between two structures (TM = 1 means a perfect match) and were calculated using TM-align [15] (updated: 15-04-2022).

Genomic analyses of phages with Z-containing DNA have revealed the existence of genes encoding enzymes involved in nucleotide metabolism [4–7]. We previously noticed the presence of a gene encoding a protein homologous to the Dut protein from *E. coli* in the genomes of the *Gordonia* phage Ghobes and the *Arthrobacter* phage Wayne (Fig. 1A) [7]. In *E. coli*, Dut is a trimeric 152 amino-acid deoxyuridine 5′-triphosphate diphosphatase (dUTPase) that catalyzes the hydrolysis of dUTP into dUMP, maintaining a low intracellular concentration of dUTP and providing dUMP to the thymidylate synthase for thymidylate (dTMP) conversion and final dTTP biosynthesis [34, 35].

Analysis of the domain architecture of the Dut-like of the *Arthrobacter* phage Wayne highlighted an N-terminal amino acid extension of 177 amino acids with no sequence similarity with sequence databases (Fig. 1B). We used AlphaFold2 [13] to predict the structure of the N-terminal part. The N-terminal structure is –145 amino-acids and adopts an overall α/β-twist fold with a five-stranded approximately β-sheet. A flexible linker connects the N-terminal and the dUTPase domains (Fig. 1B). We searched for similar structures in the PDB [25] using FoldSeek [36]. The three best matches are crystal structures of 2′-deoxynucleoside 5′-phosphate N-hydrolase enzymes from *R. norvegicus* (Rcl or DNPH1) [37], *Homo sapiens* (DNPH1) [38] and *Streptomyces rimofaciens* (MilB) [39]. These three enzymes catalyze the hydrolysis of deoxy/ribonucleoside monophosphates into free nucleobase moieties and deoxyribose/ribose 5-phosphates [39, 40]. The four next hits are 2-deoxyribosyltransferase structures from *Bacillus psychrosaccharolyticus, Desulfotalea psychrophila* (LSv54), *Enterococcus faecalis* (V583), and *Lactobacillus leichmannii*. These enzymes catalyze the direct transfer of the deoxyribosyl moiety from a purine (or a pyrimidine) deoxyribonucleoside to a purine (or pyrimidine) base (Supplementary Table 2). These enzymes are all members of the nucleoside deoxyribosyltransferase (NDT) family that contains either hydrolases or transferases with different substrate specificities [41]. Despite a low sequence identity, both types of enzymes have a similar mode of action. Transferases catalyze the reversible transfer of the 2′-deoxyribose moiety from a deoxynucleoside (dN) donor to an acceptor nucleobase (N’) (dN + N’≤> dN’+ N). Hydrolases of the NDT superfamily are enzymes that use a water molecule as acceptor and thus hydrolyze deoxyribonucleoside monophosphate (dNMP) or ribonucleoside monophosphate (rNMP) to form the free nucleobase (N) and 2′-deoxyribose 5-phosphate or ribose 5-phosphate. While the natural substrates of transferases are dN, the hydrolases, characterized so far, are specific for dNMPs or rNMPs.

We detected a similar NDT-like encoded gene in the *Gordonia* phage Ghobes genome but encoded in a separated gene (Fig. 1A). Alongside with the Dut and NDT-like proteins, the genome of *Arthrobacter* phage Wayne encodes a DUF5664 domain-containing protein (Fig. 1A). In *Acinetobacter* phage SH-Ab 15 497, the DUF5664 domain-containing protein is in tandem with a DUF550 domain and exhibits dGTP and dATP pyrophosphohydrolase activity, converting dGTP/dATP to pyrophosphate and dGMP/dAMP [4]. This suggests that DUF5664 of *Arthrobacter* phage Wayne could be involved in nucleotide pool regulation.

### Dut-like and DUF5664 enzymes are specialized in the hydrolysis of dATP and dGTP

Genomic DNAs from bacteriophages Wayne and Ghobes were amplified to clone their homologous *dut* and *ndt-*like genes. We overexpressed in *E. coli* and purified the 307-amino acid NDT-Dut-like protein from the Wayne bacteriophage and the two distinct Dut and NDT homologous proteins from the Ghobes bacteriophage (Supplementary Fig. S2). The activity of these three enzymes was tested on various nucleoside triphosphates substrates (1 mM) and the reaction products were analyzed by Liquid Chromatography - Mass Spectrometry (LC-MS) after a 30 min incubation.

**Figure 2.**
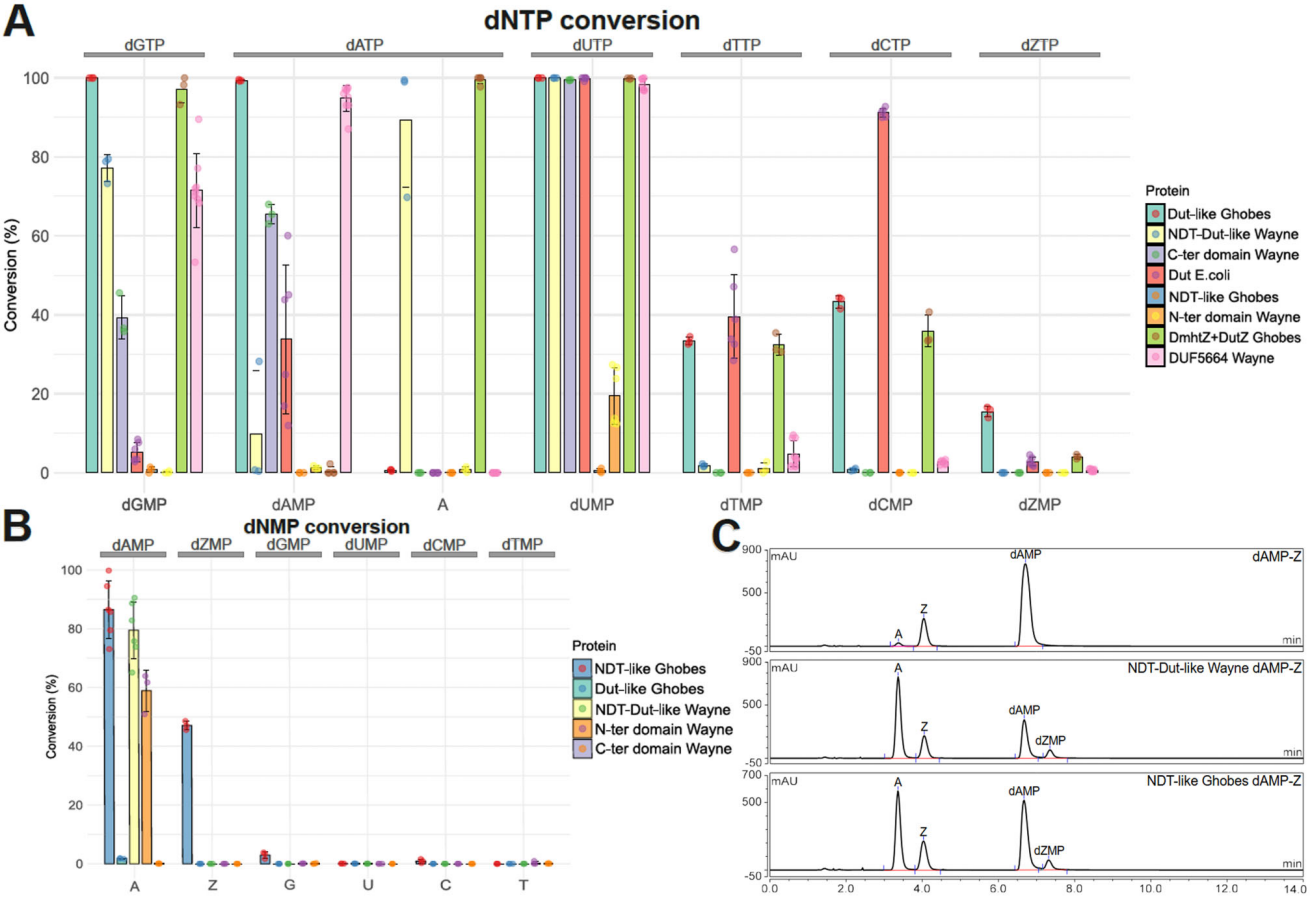
Activity and substrate specificity of PurZ0-phage enzymes. **(A)** Conversion of dNTPs (1 mM) into dNMPs or nucleobases (N for G, A, U, C, Z) by PurZ0-phage enzymes. Substrates are indicated at the top of the graph and their resulting reaction-products at the bottom. Reactions products were analyzed using LC-HRMS or UHPLC-UV for DUF5664 Wayne and their concentrations were estimated by comparison of their calculated areas with the mean spike area of each standard at 1 mM, the UV responses of these different compounds having been verified as similar. The proportions of the various products were then calculated by setting the total products formed to 100%. All potential reaction products were dNTPs, dNMPs, and adenine **(A)** in the case of hydrolysis of dATP. The error bars represent standard deviations of at least three independent experiments. **(B)** Hydrolysis of dNMPs into nonphosphorylated bases by PurZ0-phage enzymes. Reactions were analyzed using LC-MS. Product concentrations were estimated similarly by comparison with the mean spike area of each product at 1 mM, and their proportions were calculated relative to the total products formed, set to 100%. The error bars correspond to the standard deviation calculated for at least three independent experiments. **(C)** Representative UHPLC-UV chromatograms of dAMP-Z transfer reactions, with no enzyme, NDT-Dut-like enzyme from Wayne bacteriophage or NDT-like enzyme from Ghobes bacteriophages.

The Ghobes Dut-like protein led to the complete hydrolysis of dATP and dGTP into dAMP and dGMP, respectively, revealing the expected diphosphatase activity (Fig. 2A). In the presence of Wayne fused NDT-Dut-like enzyme, dGTP was also converted to dGMP. In contrast, dATP was predominantly hydrolyzed into the nonphosphorylated adenine (Fig. 2A). The production of the two separate domains of Wayne protein as two independent proteins (Supplementary Fig. S2) confirmed the role of the C-terminal domain as the catalytic unit responsible for hydrolyzing dATP into dAMP (Fig. 2A). The C-terminus part of Wayne and the Ghobes Dut both completely hydrolyze dUTP to dUMP. The Ghobes Dut enzyme also partly removes two phosphate moieties of dTTP (30%) and dCTP (40%) (Fig. 2A). The substrate specificities of the *E. coli* Dut protein were found to be different from those of bacteriophages (dUTP > dCTP > dTTP > dATP > dGTP > dZTP; Fig. 2A), emphasizing the specialization of these bacteriophage enzymes for activities related to phage propagation. No phosphatase activity was observed in the presence of ribonucleotide triphosphates regardless of the enzymes (Supplementary Fig. S3).

The DUF5664 protein, found exclusively encoded in the Wayne genome, is fused with the DUF550 protein in the PurZ *Acinetobacter* bacteriophage SH-Ab 15 497, which suggests a role in nucleotide pool regulation. Analysis of the reaction products obtained in the presence of dNTPs and DUF5664 revealed a phosphohydrolase activity converting dNTPs into dNMPs specific for dATP, dUTP and dGTP (Fig. 2A), very similar to the activity of Dut.

To determine the substrate specificity, kinetic parameters were derived from a colorimetric assay that measures inorganic phosphate release. Both the Ghobes Dut-like and Wayne NDT-Dut-like enzymes exhibited sigmoidal kinetics, fitting to the Hill model (*v* = (*V*max S*^n^*)/(*S*_50_*^n^* + S*^n^*). *S*_50_ is the substrate concentration showing half-maximal velocity, *n* is the Hill coefficient, and *V*max is the maximal velocity) (Supplementary Fig. S4, panels 1–6). This behavior, which suggests a cooperative substrate binding, was visually confirmed by the nonlinearity observed in the Eadie–Hofstee representation (*v* versus *v*/[S] (Supplementary Fig. S4, panels 1′–6′). Analysis of the apparent catalytic constant revealed a consistent tenfold preference for dATP over dGTP for both Dut enzymes (Table 1). Notably, the Dut-like enzyme from Ghobes showed its highest apparent *k*_cat_ with dATP while NDT-Dut-like from the Wayne bacteriophage still favored dUTP, the original substrate of this enzyme family (Table 1). The DUF5664 enzyme displayed a hyperbolic (Michaelis-Menten) kinetics with dATP (Supplementary Fig. S4, panels 7 and 7′) but sigmoidal (cooperative) kinetics with dGTP (Supplementary Fig. S4, panels 8 and 8′). Despite this mechanistic variability, the DUF5664 enzyme also demonstrated a tenfold higher *k*_cat_ for dATP compared to the apparent *k*_cat_ for dGTP (Table 1). However, its overall catalytic conversion rate remains lower than that of the Dut enzymes.

**Table 1.** Kinetics parameters of Ghobes Dut-like, Wayne NDT-Dut-like, and Wayne DUF5664 enzymes

		Non-Michaelian parameters			Michaelian parameters		
Enzyme	Substrate	*S* ^50^ (µM)	*k*_cat app_ (s^−1^)	n	*k*_cat_ (s^−1^)	*K*_m_ (µM)	*k*_cat_/*K*_m_ (M^−1^.s^−1^)
Dut-like Ghobes	dATP	18.2 ± 2.27	12.86 ± 0.77	1.52 ± 0.21			
	dGTP	30.2 ± 2.02	1.36 ± 0.06	3.25 ± 0.71			
	dUTP	13.1 ± 1.08	4.48 ± 0.21	1.81 ± 0.2			
NDT-Dut-like Wayne	dATP	18.8 ± 0.88	5.87 ± 0.14	2.02 ± 0.17			
	dGTP	12.7 ± 0.67	0.56 ± 0.01	2.76 ± 0.39			
	dUTP	8.5 ± 0.4	16.43 ± 0.42	2.18 ± 0.24			
DUF5664 Wayne	dATP				0.48 ± 0.022	20.3 ± 2.58	2.40E + 04
	dGTP	31.7 ± 0.87	0.053 ± 0.001	2.48 ± 0.1544			
	dUTP	12.5 ± 0.899	0.22 ± 0.02	1.52 ± 0.12			

Values correspond to the average of three replicates. Apparent *k*_cat_ (*k*_cat.app_) = *V*_max_/[E], [E] is the enzyme concentration)

### A novel deoxynucleoside monophosphate hydrolase and transferase enzyme

As Ghobes NDT-like protein did not exhibit catalytic activity on nucleoside triphosphates and the native Wayne protein was able to form adenine from dATP (Fig. 2A), we hypothesized that the NDT-like domain, homologous to the N-terminal homologous part of Wayne’s enzyme, might be responsible for the conversion of dAMP to A. Analysis of the reaction products obtained in the presence of deoxynucleoside monophosphates confirmed that N-ter Wayne’s enzyme was capable of specifically and exclusively converting dAMP to A while Ghobes’s converted dAMP and dZMP into their respective bases with no or residual activity on other dNMPs (Fig. 2B). We also showed that catalytic activity of both enzymes is specific to deoxynucleoside monophosphate, since no hydrolysis was detected in the presence of adenosine monophosphate (Supplementary Fig. S5). Like the native Wayne protein, the concomitant action of the two Ghobes proteins led to the conversion of dATP into A (Fig. 2A). The bifunctional protein of the Wayne bacteriophage is thus present in two independent protein domains in Ghobes, leading to the same activities on the nucleotide pool for these two bacteriophages.

These experiments revealed that the bacteriophage enzyme, classified in the NDT superfamily, has a hydrolase function. To test the possible transfer activity, we performed transfer experiments between dAMP, the substrate of bacteriophage enzymes, and the various nucleobases (G, A, T, C, and Z). Only the transfer of deoxyribose 5-phosphate (dR5P) from dAMP onto the Z base, forming dZMP and releasing A, was demonstrated for the two NDT-like enzymes of the PurZ0 bacteriophages (Fig. 2C and Supplementary Fig. S6). Quantitative analysis of this reaction revealed *in vitro* transfer rates of 20% for both enzymes (Supplementary Fig. S7).

Based on these results, we named these bacteriophage enzymes, DmtZ (deoxyadenosine monophosphate transferase) and DutZ for their NDT-like and Dut-like activities, respectively.

### Modeling of DmtZ-dAMP interactions

**Figure 3.**
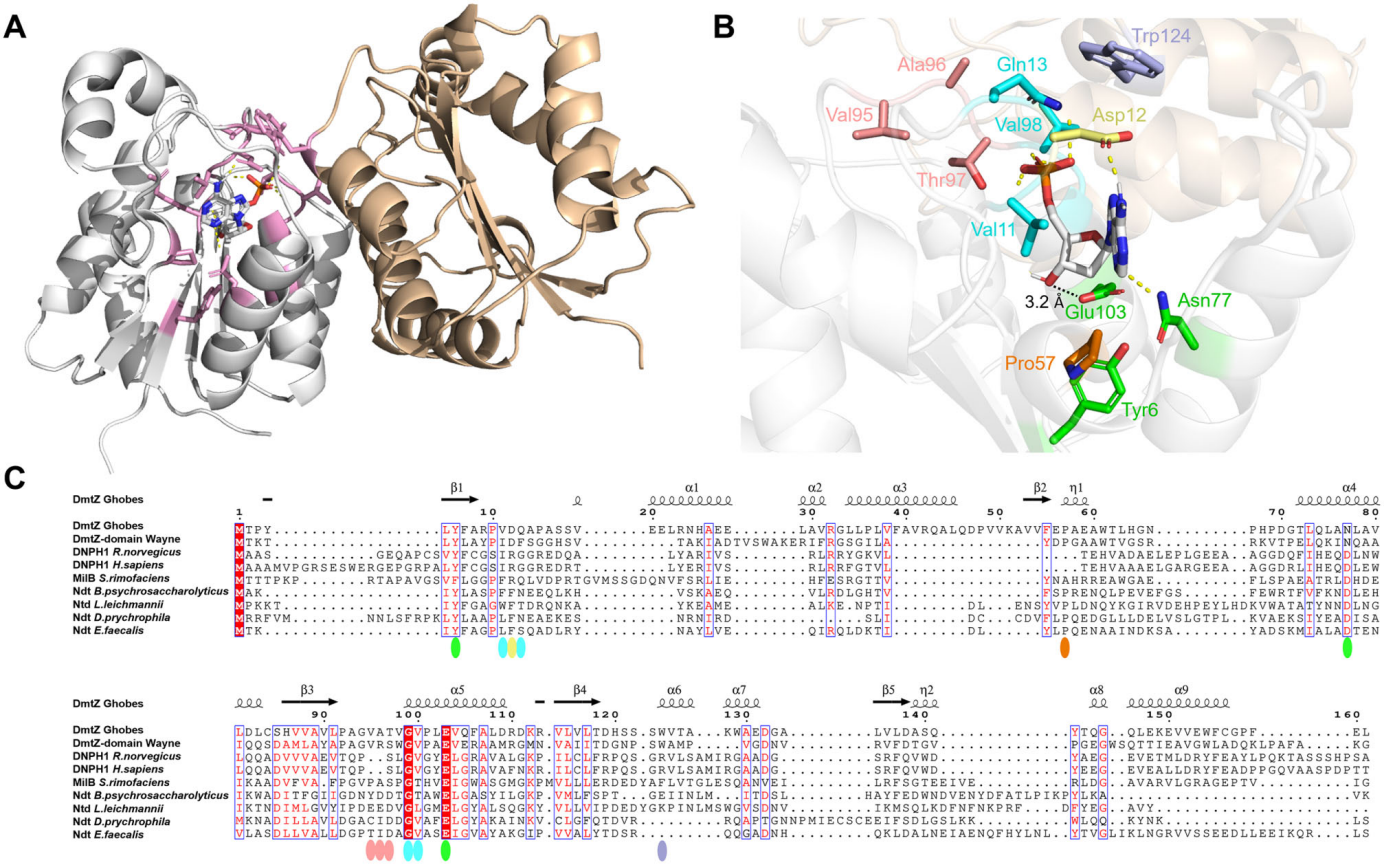
Structural insights of dAMP binding in DmtZ. **(A)** Dimeric 3D model of DmtZ dimer with one holo subunit in complex with dAMP (gray) and the other in apo form (beige). In the subunit complex with dAMP, active site residues are in pink. The protein–ligand docking model was generated by aligning an AlphaFold2-predicted DmtZ apo structure to the DNPH1 reference, transferring and replacing ligand coordinates with AutoDock Vina, and refining the lowest-energy complex using YASARA. **(B)** Zoom on the holo active site with dAMP. The active site features the catalytic triad (green), residues forming hydrogen bonds with base (yellow), and phosphate (clear blue), as well as the water-excluding Pro57 (orange), site-closing Trp124 (purple), and noncharged space-saving residues accommodating the 5′-phosphate (salmon). Distance between the Glu103 side-chain oxygen atom and the deoxyribose 3′-hydroxyl group is indicated in black. **(C)** Sequence alignment of DmtZ and their closest structural homologs. Key residues from panel (B) are indicated by colored ovals.

A dimeric model of the DmtZ protein from Ghobes, complexed with dAMP, was obtained based on the Rcl structure (PDB ID:4FYI) (Fig. 3A and B). Easily identified in DmtZ sequence alignments (Fig. 3C), the catalytic triad, characteristic of the NDT superfamily, includes the Tyr6, Asn77, and Glu103 residues (Fig. 3B). First, Glu103 is located at the same position as Glu101 in the LhNDT from *Lactobacillus helveticus*, which is known to be involved in the formation of a covalent bond with the 3′-hydroxyl group of the 2′-deoxyribose [42, 43]. In our model, this interaction is also suggested by the location of the Glu103 side-chain oxygen atom (OE2) at 3.2 Å from this 3′-hydroxyl group. Secondly, the Tyr6 position is ideal to form a hydrogen bond with Glu103, which should restrain its orientation for covalent bond formation and exclusion of ribosylated bases [39]. Thirdly, Asn77 is well placed to stabilize different catalytic states of Glu103 and the base after hydrolysis of the glycosidic bond [43, 44]. Interestingly, most NDT-hydrolase crystallographic structures feature an aspartate residue instead of an asparagine residue, but this change has already been explained as an adaptation to high pH values in the transferase TbPDT from *Trypanosoma brucei* [31].

At the top of the active site, the Trp124 residue from the second monomer appears to close the active site, as for the ionic interaction between Arg19 and Asp62 residues in Rcl from *R. norvegicus*. Therefore, Trp124 could act as a rotating lid involved in the substrate binding (Fig. 3B).

Regarding the 5′-phosphate binding pocket, DmtZ does not display aspartate or asparagine residues, known to stabilize the 5 hydroxyl group in NDTs, but harbors non-charged space-saving residues, like in hydrolases [28]. In the DmtZ model, the 5′-phosphate makes hydrogen bonds with the Val11, Gln13, Val98, and Gly99 residues, supporting the specificity for mono-phosphorylated substrates.

However, DmtZ also shares common features with transferases that are absent in hydrolases, namely the Pro57 residue, conserved in all known transferases, that is involved in protecting the active site from water molecules, theoretically excluding any hydrolase activity [46].

Regarding the dAMP specificity, the Asp12 residue could act as a hydrogen bond acceptor with the 6′-amine group of the adenine, thus preventing the binding of dGMP, which presents a 6′-ketone.

### Critical residues involved in DmtZ activity

**Figure 4.**
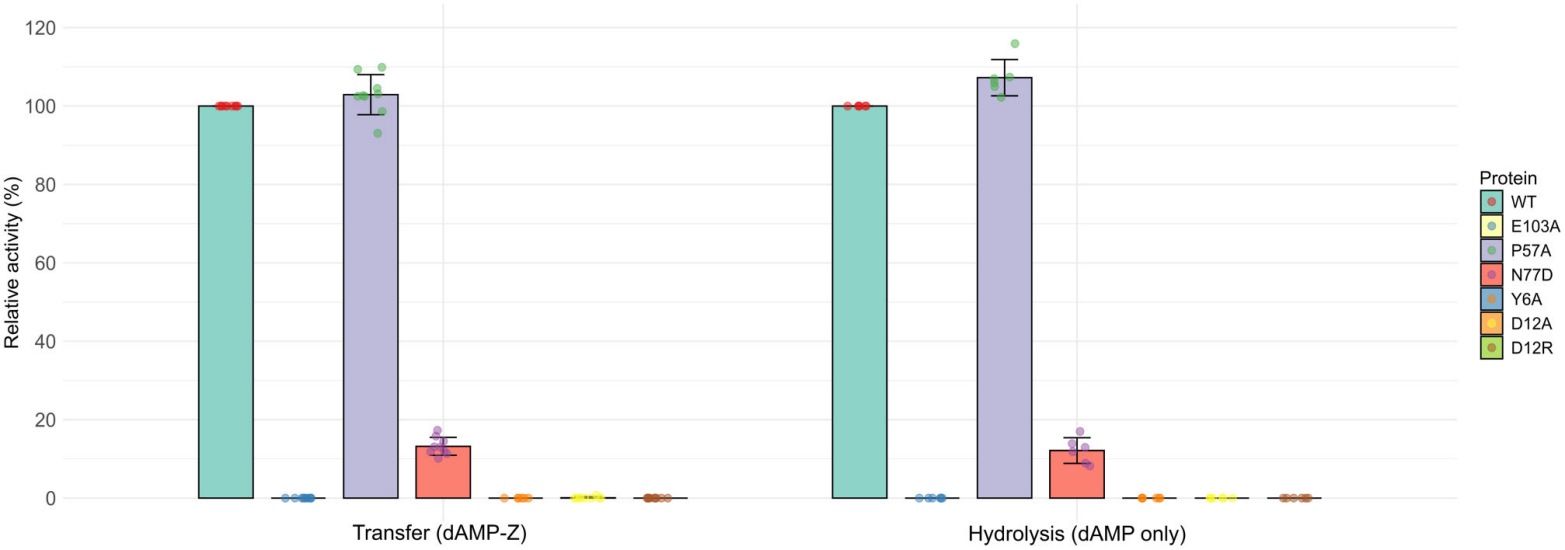
Relative transfer and hydrolysis activity of DmtZ mutants. Enzymes were incubated either with 3 mM dAMP and with 1 mM Z, or with dAMP alone and reaction products were analyzed using UHPLC-UV after 1 h of reaction. The error bars correspond to the standard deviation calculated for at least three independent experiments.

Based on our model of enzyme-substrate interactions, amino acids of the active site that may influence substrate specificity or preferentially promote one of the two activities were selected for mutagenesis. Mutant activities were determined either by spectrophotometry following the appearance of deoxyribose 5-phosphate for hydrolase activity (Table 2), or by HPLC for both activities (Fig. 4). The hydrolase activity of wild-type Ghobes DmtZ, quantified by the spectrophotometric assay, confirmed the LC-MS results with major efficiency on dAMP and residual activity on dGMP (Table 2). As expected, given their involvement in the catalytic triad, substitution of the nucleophile Glu103 or Tyr6 with an alanine abolished the hydrolase and transferase activities of DmtZ. The mutagenesis of the Asn77 to an aspartic acid, the most frequent residue found at this position in the NDT superfamily, altered the efficiency of the enzyme for its two functions, revealing its importance in the catalytic activity.

**Table 2.** Kinetic parameters for the hydrolysis of dAMP or dGMP by wild type or mutant DmtZ

		WT	E103A	P57A	N77D	Y6A	D12A	D12R
dAMP	*K*_m_ (µM)	7.2 ± 0.9	nd	25.4 ± 1.7	140 ± 11.6	nd	nd	nd
	*k*_cat_ (s^−1^)	1.9 ± 0.051	nd	0.47 ± 0.01	0.39 ± 0.01	nd	nd	nd
	*k*_cat_/*K*_m_ (s^−1^ M^−1^)	2.64E + 05	nd	3.18E + 04	2.76E + 03	nd	nd	nd
dGMP	*K*_m_ (µM)	2760 ± 99	nd	1020 ± 44.5	nd	nd	800 ± 33	nd
	*k*_cat_ (s^−1^)	4.05E-02 ± 5E-04	nd	5.18E-02 ± 5E-04	nd	nd	6.70E-03 ± 6E-05	nd
	*k_cat_/K_m_* (s^−1^ M^−1^)	1.47E + 01	nd	5.08E + 01	nd	nd	8.42	nd

nd = nondetected. Standard deviations were calculated from three independent experiments.

The Asp12 residue, which could be involved in dAMP specificity, conducted to loss of activity whatever the substitution into an alanine or an arginine, commonly found in hydrolase activities, suggesting this residue as an important contributor to DmtZ activity.

Mutation of the Pro57 residue, which may be involved in transferase activity, to alanine did not induce a change in either the transferase or the hydrolase activity when estimated by HPLC (Fig. 4). However, the establishment of kinetic parameters (Table 2) revealed a one-log decrease in the hydrolase activity.

### dutZ and dmtZ genes are enriched in PurZ0 genomes

We searched for PurZ homologs using the PurA Pfam accession PF00709 in a collection of 53 887 viruses from the NCBI. We found that phage genomes encode 481 homologous proteins to the adenylosuccinate synthetase. To sort out between 2-amino adenylosuccinate synthetases (PurZ) and bacterial adenylosuccinate synthetase paralogs (PurA), we searched for PurA homologs in a collection of 47 894 prokaryotic genomes that are representative of the genetic diversity of the prokaryotes and randomly selected up to two sequences per prokaryotic phylum (298 sequences from 160 distinct phyla). The dataset was supplemented with the PurA sequence of *E. coli* and five sequences related to PurZ previously described [12]. We built a phylogenetic tree with the 767 protein sequences. The PurA sequence of *E. coli* is placed within a monophyletic group (bootstrap: 90) of 251 sequences that comprises 246 out of the 298 prokaryotic sequences (Supplementary Fig. S8). Alignment of these sequences with the *E. coli* PurA sequence highlights a 99% conservation of the essential Asp13 residue of the adenylosuccinate synthetase.

**Figure 5.**
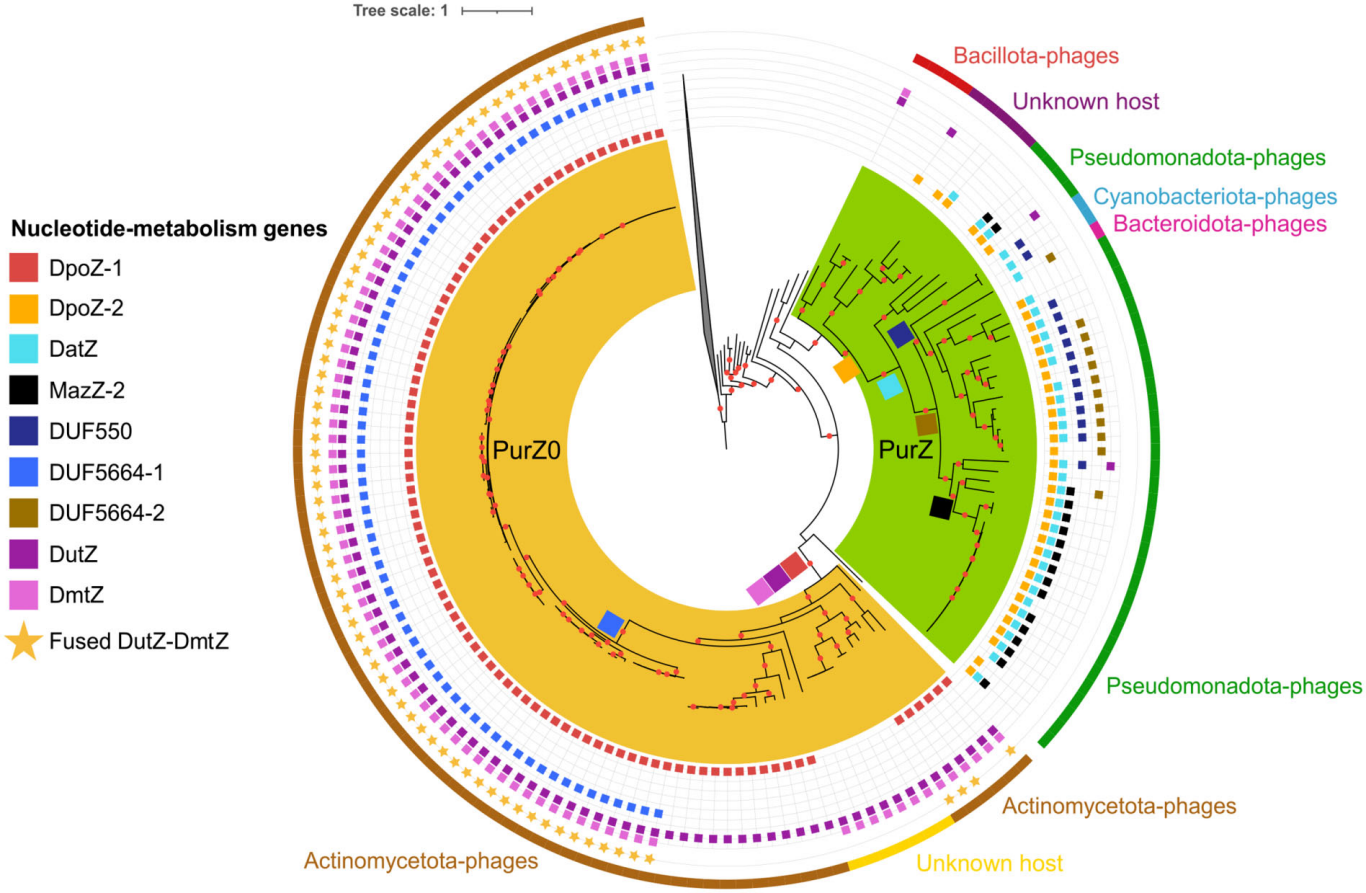
Maximum-likelihood tree of NCBI viral PurZ homologs. The tree has been constructed using the LG + G4 model. The root corresponds to other PurA homologs. Red points indicate bootstrap values ≥90. The vConTACT2-based clusters on the external band correspond to clusters formed using vCONTACT2 (Supplementary Fig. S9). Squares on tree branches represent gene acquisition events.

The five PurZ-related sequences cluster along with the PurZ sequences and together comprise 151 sequences that refer to the PurZ group (bootstrap: 100). These include four archaeal sequences that have a Ser or a Gly residue in the catalytic site whereas the aspartic acid is replaced by a serine residue in 100% of the 147 remaining phage sequences. PurZ sequences from phages are divided into two monophyletic subgroups named PurZ0 (bootstrap: 100) and PurZ (bootstrap: 100) and show preferences for ATP and GTP, respectively. While the four archaeal sequences are basal to these two subgroups, their positions are not supported by strong bootstrap values (Fig. 5). We mapped the presence/absence of DmtZ and DutZ encoded proteins into the phylogenetic tree. Every 98 PurZ0 encoding genomes have a *dutZ* gene, 85 of them also share a *dmtZ* gene, and 77 out of 85 are fused with a *dutZ* gene (Fig. 5).

### Z-containing DNA phages are genetically, phylogenetically and ecologically diverse

**Figure 6.**
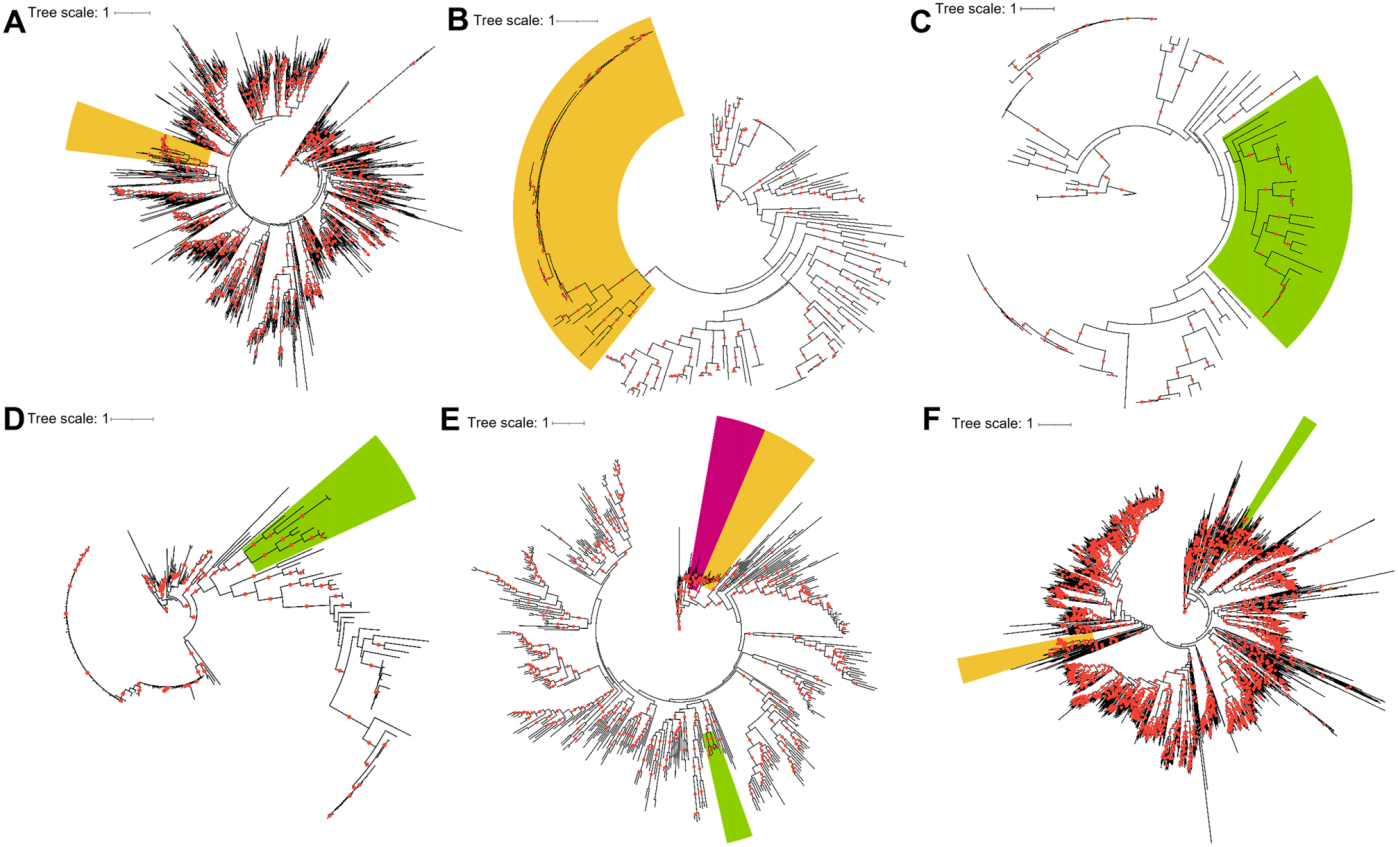
Phylogenies of Z-containing DNA synthesis proteins. **(A)** Maximum-likelihood tree of NCBI viral dUTPase constructed with the model LG + R9. Proteins from PurZ-containing phages are shown in green, while those from PurZ0-containing phages are in yellow. Red points indicate bootstrap values ≥90. **(B)** Maximum-likelihood tree of NCBI viral NDT homologs constructed with the model LG + R5. **(C)** Maximum-likelihood tree of NCBI viral DatZ-like constructed with the model LG + R5. **(D)** Maximum-likelihood tree of NCBI viral DUF550 homologs constructed with the model VT + R5. **(E)** Maximum-likelihood tree of NCBI viral DUF5664 constructed with the model Blosum62 + R7. *Arthrobacter* phages that do not contain a PurZ gene are shown in purple. **(F)** Maximum-likelihood tree of NCBI viral DNA polymerase I constructed with the model LG + F + R10.

Whereas genomes from PurZ0 encoding phages have *dutZ, dmtZ*, and*DUF5664* genes to regulate their nucleotide pool, phages encoding the *purZ* variant gene mostly encode a DatZ which hydrolyses dATP into dA [6] and either a MazZ-2 (a distant homolog of MazZ which hydrolyses dGTP into dGMP) [8] or a DUF550 (same function as MazZ) [4] often fused with a DUF5664 domain (Fig. 5). Phylogenetic analyses of DutZ, DmtZ, DatZ, and DUF550 reveal that each of these enzymes forms a monophyletic group (Fig. 6A–D), suggesting a single evolutionary origin for each within Z-containing DNA phages. DUF5664 domain-containing proteins are encoded in both PurZ and PurZ0 genomes. However, phylogenetic reconstruction of DUF5664 reveals two distinct monophyletic groups that fit the PurZ0/PurZ separation [bootstraps 99 for PurZ0 (DUF5664-1) and 93 for PurZ (DUF5664-2) (Fig. 6E)], suggesting two independent acquisitions. Interestingly, the DUF5664-1 variant is encoded in PurZ0-phages that infect *Arthrobacter* bacteria and, phylogenetically, these phage proteins are placed next to DUF5664-like proteins from canonical-genome *Arthrobacter* phages, suggesting a vertically inheritance of the DUF5664-1 gene by PurZ0 genomes from *Arthrobacter* phages.

Similarly, our phylogenetic analysis of viral DNA polymerase I, which included DpoZ proteins, corroborated the recent finding that DpoZ proteins cluster into two distinct phylogenetic groups (100% bootstrap value; Fig. 6F) [47]. Significantly, within our analysis, the DNA polymerase I sequences perfectly aligned with the PurZ0 (DpoZ-1) and PurZ (DpoZ-2) classification. This strong congruence provides further evidence for two independent acquisitions of a DNA polymerase I that preferentially selects 2-aminoadenine over adenine in viruses.

In parallel, construction of a gene-sharing network to infer relationships of Z-containing DNA phages with other phages show that Z-containing DNA phages comprise at least seven phylogenetically distinct groups (Supplementary Fig. S9). Two out of seven are PurZ0-encoding phages whereas the remaining five groups comprise PurZ-encoding phages (Fig. 5). Notably, the genomes from five of these seven groups showed close connections (e.g. sharing a significant number of genes) within the network to phage genomes with cannonical DNA that infect related hosts. For instance, the Z-genome of *Synechococcus* phage S-2L is closely connected with non-Z-genomes of phages infecting *Cyanobacteriota* (Supplementary Fig. S9). This diversity of hosts is corroborated by the wide environmental distribution of Z-containing DNA phages, ranging from oceanic and freshwater bodies to wastewater, soil and animal reservoirs (Supplementary Table S3).

## Discussion

In search of new potential enzymes involved in regulating the nucleotide pool of bacteriophages, we characterized three enzymatic activities from Z-containing DNA bacteriophages harboring a PurZ0-type adenylosuccinate synthase.

The DutZ and DUF5664-1 enzymes are both dNTP phosphohydrolases that catalyse the conversion of dATP to dAMP and dGTP to dGMP. Establishment of their kinetic parameters revealed that both enzymes exhibit a preference for hydrolyzing dATP over dGTP. This kinetic difference may reflect a dual functional requirement: an urgent need to deplete dATP to promote the incorporation of dZTP, and a more moderate requirement to hydrolyze dGTP (to reform dZTP) given that dGTP is essential for bacteriophage replication. The kinetic analysis of DutZ enzymes revealed allosteric enzymes, consistent with their known trimeric structure, which must play a key role in adjusting specific activities according to intracellular nucleotides fluxes. These enzymatic activities therefore contribute to the creation of a regulated cellular environment conducive to the production of the Z-genome by their specific DNA polymerase. In *purZ* encoding bacteriophages, these two functions are ensured by two independent genes, *datZ* and *mazZ* (or *DUF5664-DUF550* in *Acinetobacter phage SH-Ab 15 497)*, hydrolyzing dATP and dGTP, respectively. Genomic analyses of PurZ0 bacteriophages have shown that, in some genomes, a *dmtZ* gene is consistently located near the *dutZ* gene. These two genes encode either two independent proteins or a single fusion protein, depending on the bacteriophage.

Structural analogy with the DNPH1 protein from *R. norvegicus* [48] and identification of key residues in protein sequence alignments with NDT superfamily enzymes suggested a potential deoxynucleoside 5-monophosphate N-glycosidase activity for DmtZ. Unlike most enzymes in this superfamily, which generally exhibit purine or pyrimidine preference, DmtZ showed a main selectivity for dAMP hydrolysis on canonical dNMP and unexpected exclusive transfer activity of deoxyribose 5-phosphate from dAMP to the non-canonical Z-base. Analysis of the mutant’s activity confirmed the expected role of the catalytic triad residues, characteristic of the NDT superfamily, notably Glu103 for covalent catalysis, Tyr6 for its role in stabilizing Glu103 and Asn77 found in few transferases at this position. Highlighted in the DmtZ-dAMP 3D model, the aspartic residue at position 12 could be a main contributor to substrate binding and catalysis. Future structural determination of DmtZ in complex with ligands will elucidate the precise roles of various interacting and catalytic residues, as well as the subtleties of this enzyme’s dual activity.

**Figure 7.**
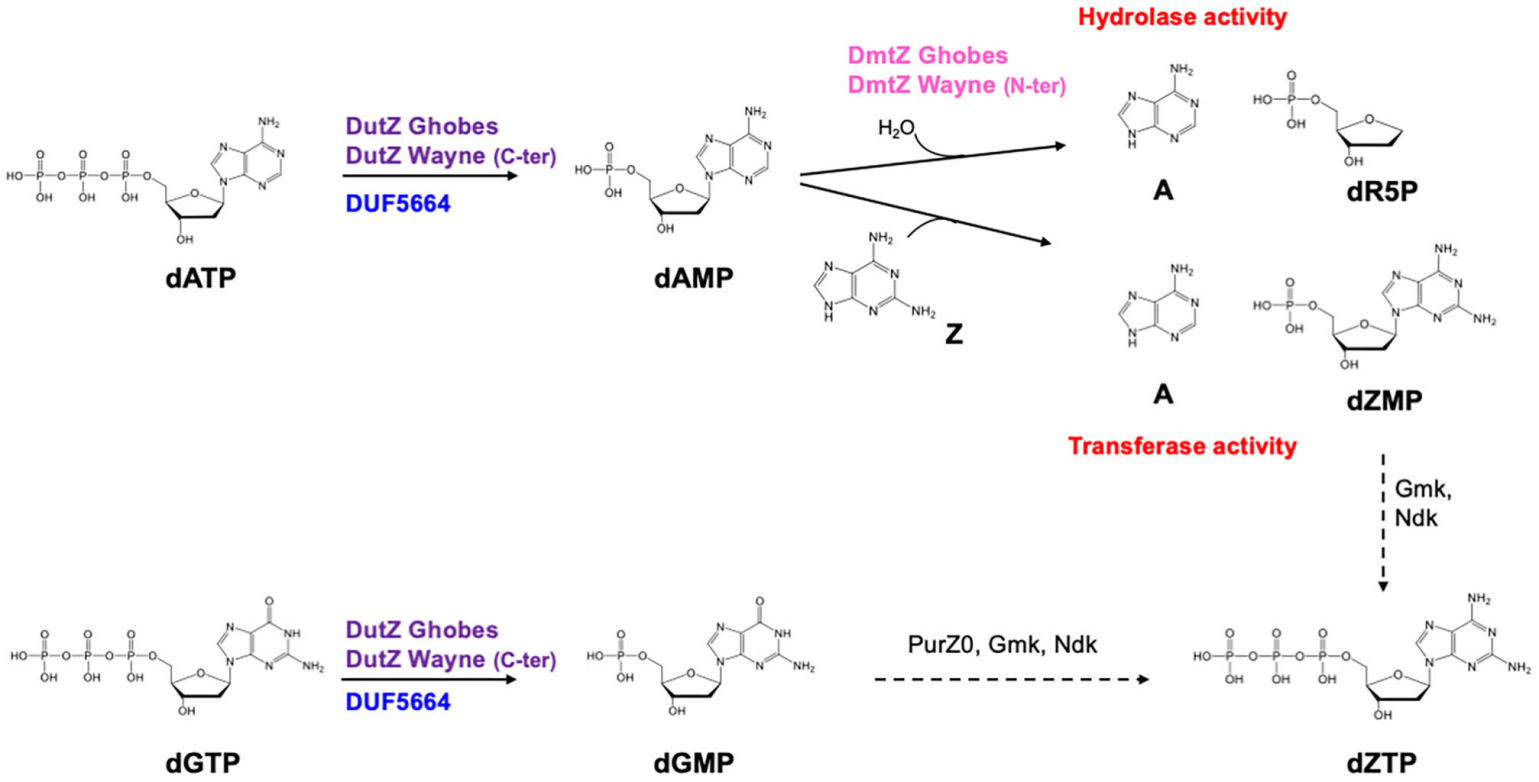
A new route for dZTP synthesis in PurZ0 bacteriophages. Reactions catalysed by DutZ, DmtZ from Ghobes bacteriophage, DUF5664-1 and DmtZ-DutZ enzymes from Wayne bacteriophage. Dashed arrows indicate reactions catalyzed by host enzymes or uncharacterized phage proteins and intermediate compounds are detailed in Supplementary Fig. S1.

Kaminski and Labesse constructed synthetic 2′-deoxyribose 5-phosphate transferase variants [41], which exhibited structural characteristics of both transferases and hydrolases. However, these engineered enzymes were functionally characterized as transferase, showing no hydrolase activity. Consequently, no enzyme, natural or synthetic, combining hydrolase and transfer specificities, has been observed prior to DmtZ, which also represents the first natural 2′-deoxyribose 5-phosphate transferase. The transfer capability of DmtZ, combined with the dATP hydrolysis activity of DutZ or DUF5664-1, provides the bacteriophage with a second pathway for dZTP biosynthesis (Fig. 7) and a mechanism to salvage Z bases potentially generated from bacteriophage DNA cleavage by bacterial enzymes.

Our comparative genomic and phylogenetic analyses illuminate the complex evolutionary history of the Z-containing DNA propagation system in phages. Indeed, while the core purZ gene has a single origin with subsequent diversification into purZ0 and purZ, the overall system has been shaped by multiple evolutionary events.

The distinct genetic landscapes observed in PurZ0- and PurZ-encoding phages, particularly the contrasting sets of nucleotide pool regulating enzymes (DutZ/DmtZ/DUF5664-1 in PurZ0 vs. DatZ/MazZ-2/DUF550 alone (MazZ) or fused with DUF5664-1 in PurZ), suggest that these lineages have evolved distinct strategies for managing their nucleotide pools. The divergent evolutionary trajectories are further exemplified by the DUF5664-1 domain. The strong phylogenetic separation of DUF5664-1 (found in PurZ0 phages) and DUF5664-2 (often fused with DUF550 in PurZ phages) indicates two independent origins. Interestingly, the phylogenetic proximity of DUF5664-1 in *Arthrobacter*-infecting PurZ0 phages to DUF5664-like proteins from *Arthrobacter* phages harboring a canonical genome suggests a potential vertical, rather than horizontal inheritance of this specific domain within this lineage. Further evidence for independent evolutionary paths within the Z-containing DNA system comes from the DNA polymerase I. The phylogenetic clustering of this enzyme from Z-containing DNA phages into two distinct groups, aligning with the PurZ0 and PurZ separation (DpoZ-1 and DpoZ-2, respectively), suggests that the ability to preferentially utilize 2-aminoadenine over adenine has evolved on two separate occasions.

The broader context provided by the gene-sharing network analysis (Supplementary Fig. S9) underscores the dynamic nature of Z-containing DNA phage evolution. The presence of at least seven distinct groups within Z-containing DNA phages highlights the diversity of this trait. Notably, the close connections observed between five of these groups and phages with canonical DNA infecting related hosts strongly imply that the capacity to synthesize Z-containing DNA has not been exclusively maintained through vertical inheritance. Instead, these connections suggest that horizontal gene transfer has likely played a significant role in the acquisition and distribution of the Z-containing DNA synthesis machinery across different phage lineages.

Considering the challenge of propagating a noncanonical DNA structure, our findings indicate that different phage lineages present in diverse ecosystems have arrived at functionally similar solutions through distinct evolutionary paths—a hallmark of functional convergence. While the *purZ* gene provides the central enzymatic activity and traces back to a single origin, the accompanying machinery for nucleotide pool manipulation and DNA replication shows evidence of independent origins and dissemination via horizontal gene transfer. This suggests that the selective pressure to maintain or utilize Z-containing DNA has driven the independent assembly of functionally coherent systems from diverse genetic building blocks.

## Supplementary Material

gkag079_Supplemental_File

## Data Availability

Generated HMMs used for MazZ-2 and Ndt-like mining and Fasta of protein sequences used to build phylogenetic trees have been uploaded the Figshare: https://doi.org/10.6084/m9.figshare.29087063.v1

## Notes

Present address: Alderys, Orsay 91400, France
