# Correction to ‘Functional convergence in Z-DNA biosynthesis highlighted by the characterization of nucleotide metabolism enzymes in bacteriophages’

**DOI:** 10.1093/nar/gkag563

**Published:** 2026-05-28

This is a correction to: Florent Poubanne, Ekaterina Darii, Aline Mariage, Eddy Elisée, Peggy Sirvain, Camille Hassan, Julie Rivollier, Aurélie Fossey-Jouenne, Alain Perret, Raphaël Méheust, Valérie Pezo, Functional convergence in Z-DNA biosynthesis highlighted by the characterization of nucleotide metabolism enzymes in bacteriophages, Nucleic Acids Research, Volume 54, Issue 4, 27 February 2026, gkag079, https://doi.org/10.1093/nar/gkag079

Following publication of this article, we received correspondence highlighting potential ambiguity in our use of the term “Z–DNA.” The concern raised relates to the longstanding distinction between Z–DNA as a left–handed DNA helical conformation and the use of “Z” to denote a modified nucleotide base (2-aminoadenine) incorporated into DNA sequences. We appreciate that this terminology has been a source of confusion.

The abstract clearly describes the intended meaning of “Z–DNA” as referring to DNA containing the modified base Z. However, to avoid any possible misunderstanding for readers, and in line with efforts within the community to distinguish more clearly between structural and compositional uses of the term, we have made corrections throughout the article.

Specifically, all instances of “Z–DNA,” including in the title, have been amended to “Z-containing DNA”.

We thank the correspondents for drawing attention to this issue.

This change does not affect the results, discussion and conclusions presented in the article. The published article has been updated.

