# Supplementary material for: Functional convergence in Z-containing DNA biosynthesis highlighted by the characterization of nucleotide metabolism enzymes in bacteriophages
Source: Nucleic Acids Res. 2026 Feb 9;54(4):gkag079. doi: 10.1093/nar/gkag079 (PMC12884076; doi:10.1093/nar/gkag079)
Supplement: gkag079_Supplemental_File [file gkag079_supplemental_file.pdf]

Functional convergence in Z-DNA biosynthesis highlighted by the characterization of nucleotide metabolism enzymes in bacteriophages

Florent Poubanne[1], Ekaterina Darii[1], Aline Mariage[1], Eddy Elisée[1], Peggy Sirvain[1], Camille Hassan[1], Julie Rivollier[1,2], Aurélie Fossey-Jouenne[1], Alain Perret[1], Raphaël Méheust[1*] and Valérie Pezo[1*]

1, Génomique Métabolique, Genoscope, Institut François Jacob, CEA, CNRS, Univ Evry, Université Paris-Saclay, 2 rue Gaston Crémieux, 91057 Evry, France

2, TESSSI, 81 rue Réaumur, 75002 Paris, France

*To whom correspondence should be addressed. Tel +33 1 60 87 45 60, Email: vpezo@genoscope.cns.fr
Correspondence may also be addressed to Raphaël Méheust, Email: raphael.meheust@genoscope.cns.fr

Present address: Eddy Elisée, Julie Rivollier, Alderys, Orsay, 91400, France

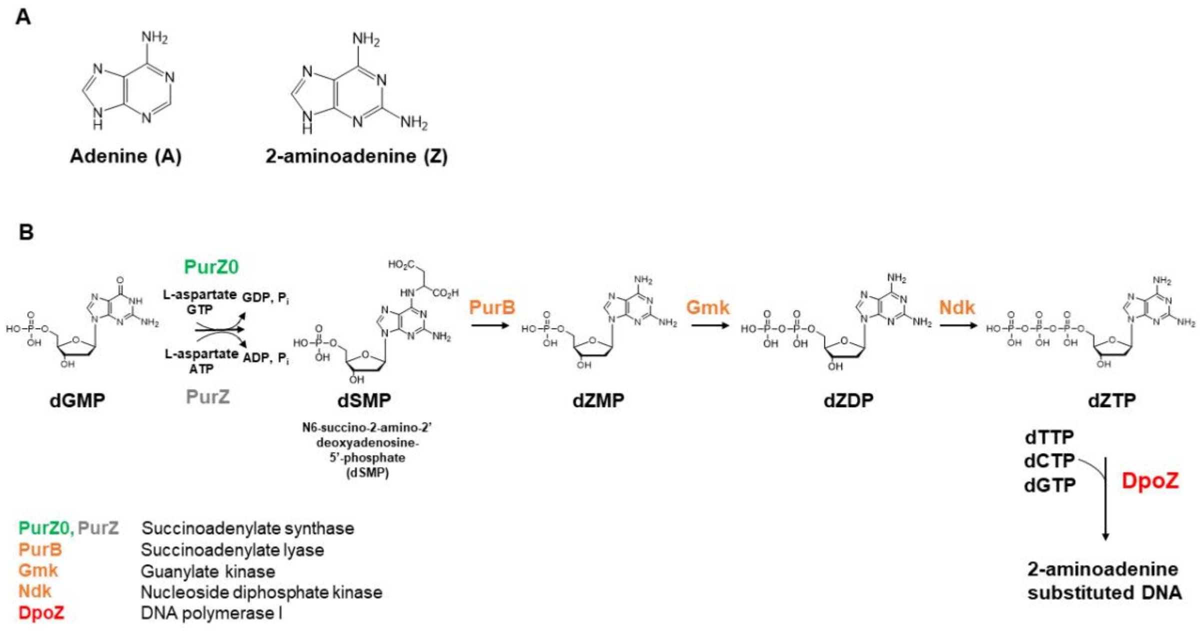

Supplementary Figure 1. Biosynthetic pathway of Z-DNA biosynthesis. **(A)** Adenine and 2-aminoadenine. **(B)** Steps, enzymes and intermediates compounds of Z-DNA biosynthesis. PurZ or PurZ0 and DpoZ are encoded by bacteriophages. PurB, Gmk and Ndk are encoded by bacterial hosts or uncharacterized phage proteins.

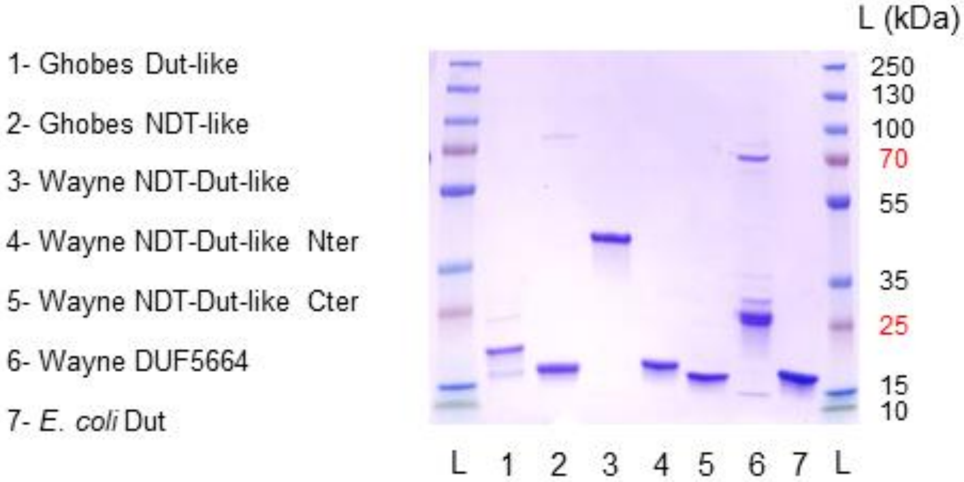

Supplementary Figure 2. SDS PAGE gel analysis of recombinant proteins purified in this study. Lane L: Molecular weight marker. 1µg of each recombinant protein was loaded.

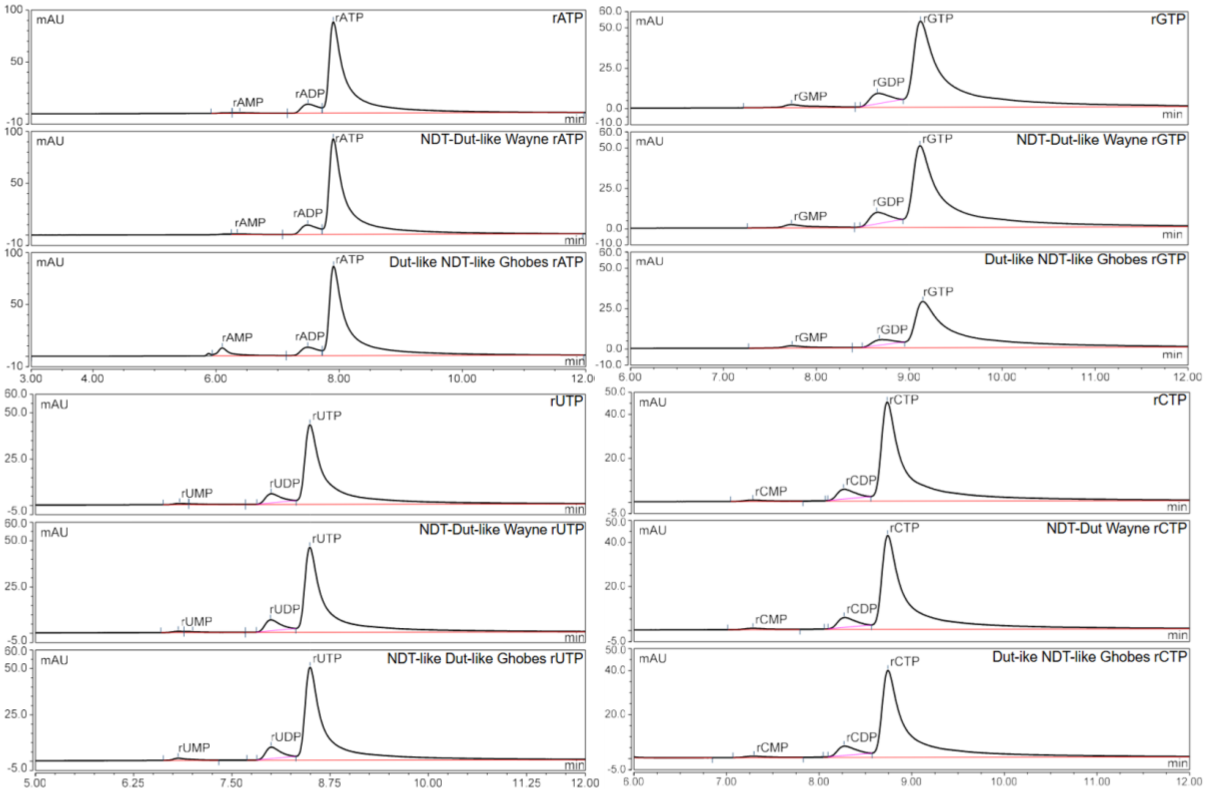

Supplementary Figure 3. Representative UHPLC-UV chromatograms of rNTP hydrolysis reactions with or without enzymes. Dut-like and NDT-like Ghobes were co-incubated.

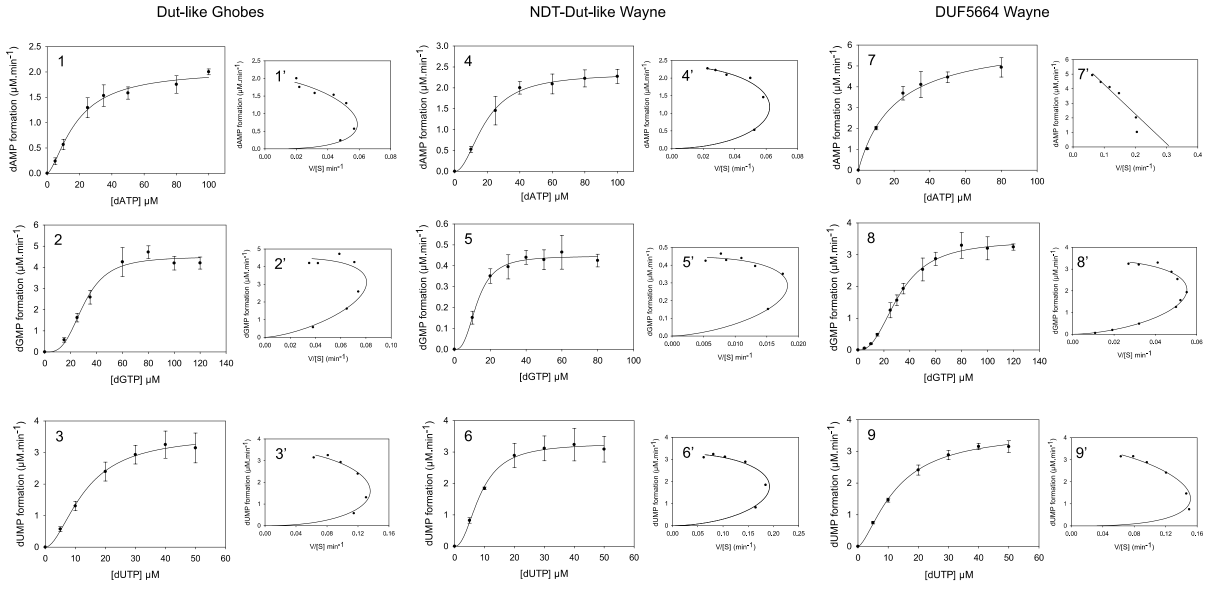

Supplementary Figure 4. Determination of enzyme kinetics parameters. Kinetic assays of Ghobes Dut-like, Wayne NDT-Dut-like and Wayne DUF5664-1 enzymes. Panels A1 to A9: Rates of dNMP formation versus concentration of dNTP. Curves were drawn using Sigma-Plot software. The Michaelis–Menten equation $v = (V_{max}\ S)/(K_m + S)$ applies for plot A7, and the Hill equation $v = (V_{max}\ S^n)/(S_{50}^n + S^n)$ applies for all other plots. S_{50} is the substrate concentration showing half- maximal velocity, n is the Hill coefficient, and V_{max} is the maximal velocity. Panels A1' to panel A9' correspond to Eadie-Hofstee representation (v versus v/[s]) of the kinetics which is linear for Michaelis Menten kinetics (panel 7') and nonlinear for cooperative enzymes (panel 1', 2', 3', 4', 5', 6', 7' and 9').

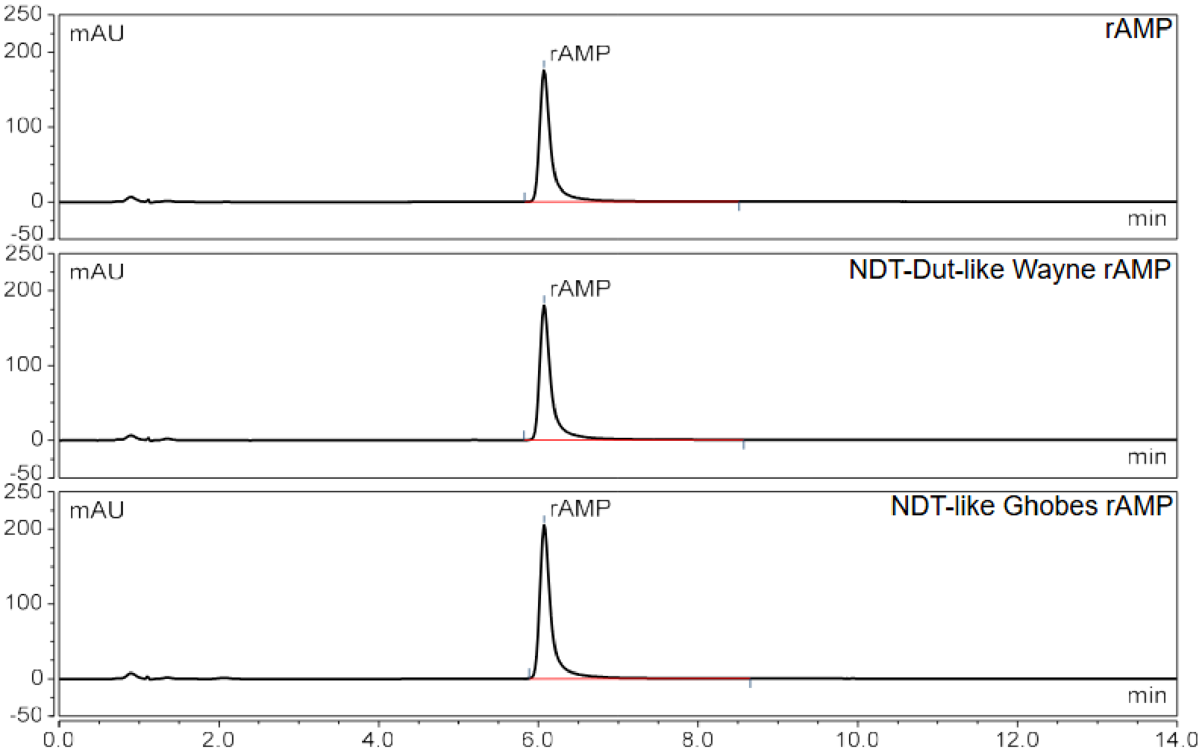

Supplementary Figure 5. Representative UHPLC-UV chromatograms of rAMP hydrolysis with or without enzymes.

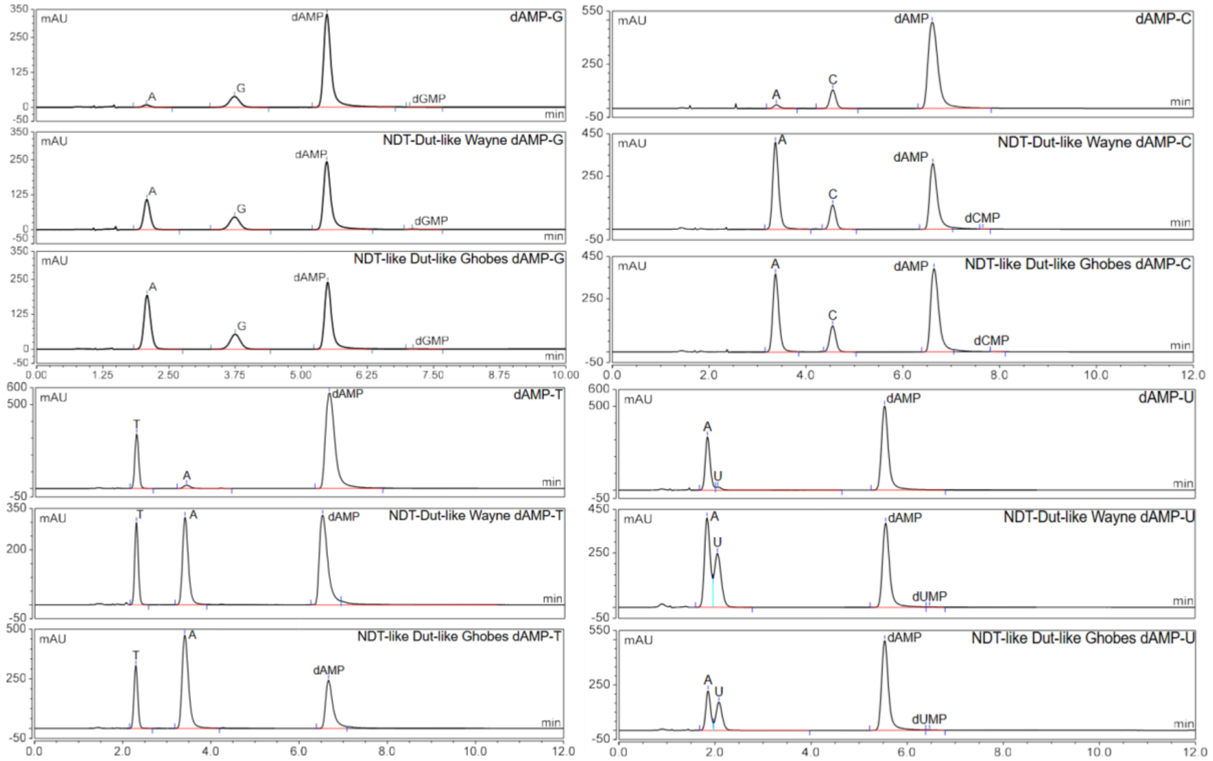

Supplementary Figure 6. Representative UHPLC-UV chromatograms of dAMP-N transfer reactions with or without enzymes.

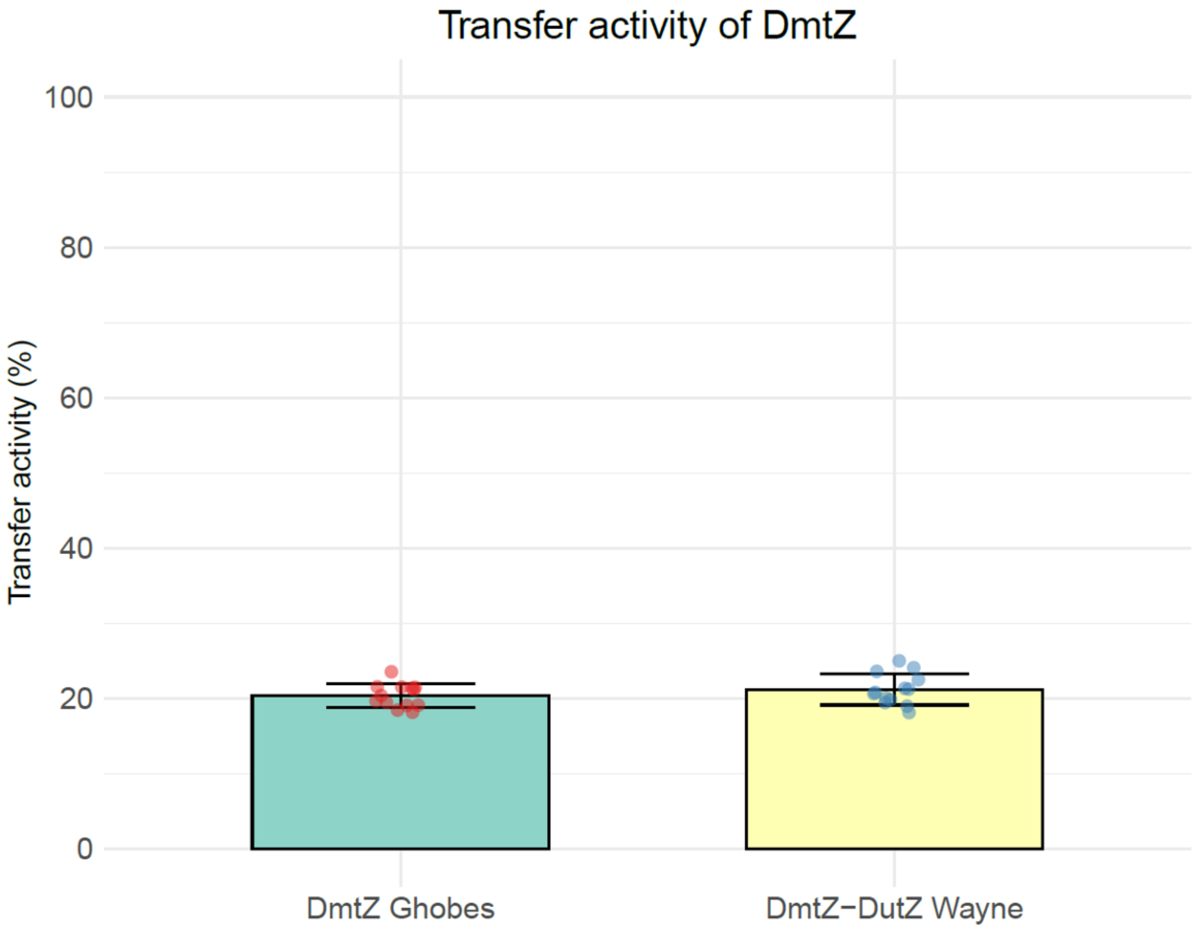

Supplementary Figure 7. Transfer activity of DmtZ from Ghobes and DmtZ-DutZ from Wayne bacteriophages. Enzymes were incubated with dAMP at 3 mM and Z at 1 mM and products were analyzed using UHPLC-UV. The concentration of dZMP was estimated by comparison with the average surface area of the dZMP peak at 1 mM, and its proportion was calculated relative to the total amount of Z and dZMP formed, set to 100%. The error bars correspond to the standard deviation calculated for at least three independent experiments.

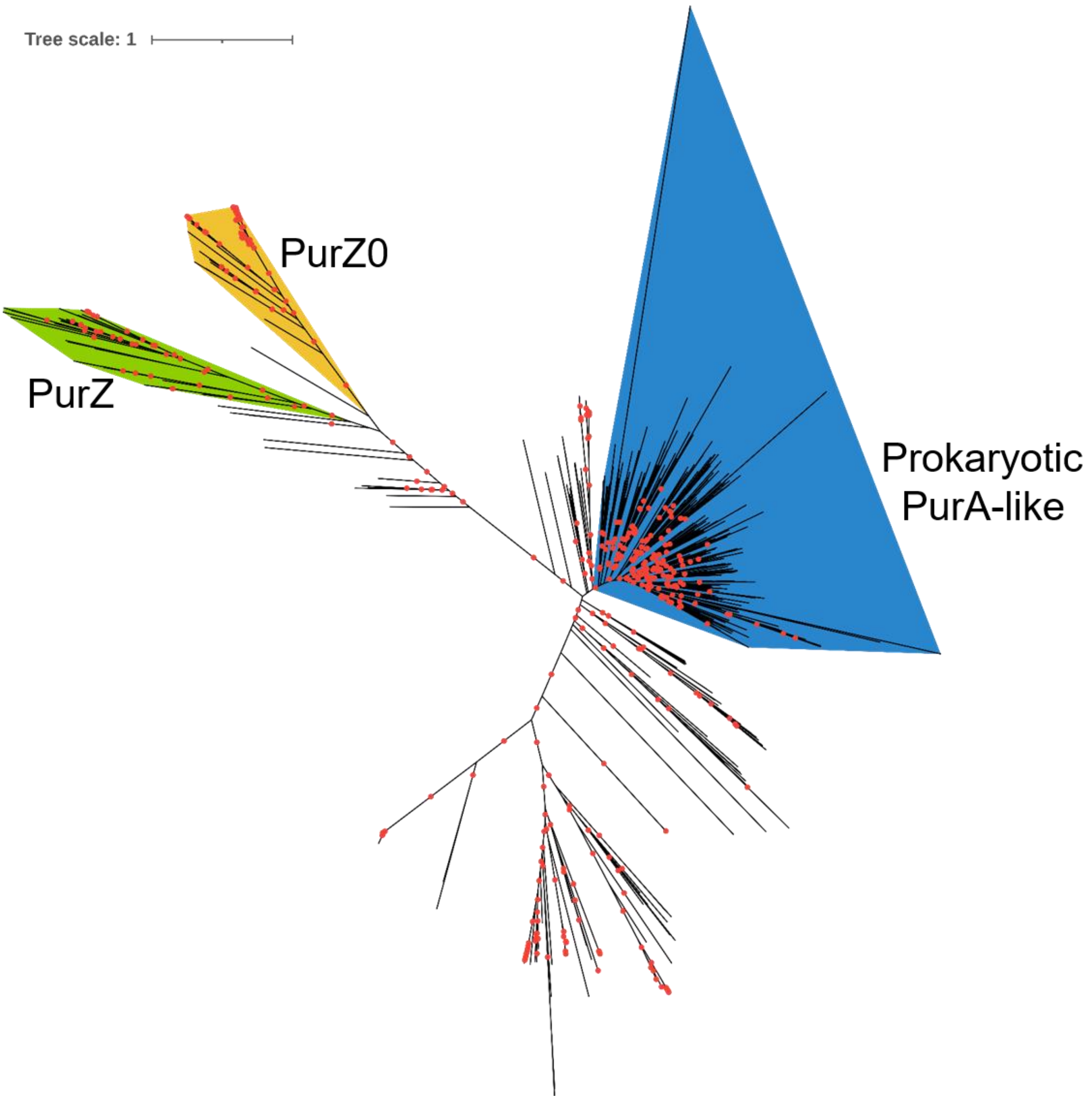

Supplementary Figure 8. Unrooted maximum-likelihood tree of PurA-like proteins. The tree has been constructed using the LG+G4 model. Proteins from PurZ-containing phages are shown in green, while those from PurZ0-containing phages are in yellow and 251 prokaryotic PurA-likes are in blue. Red points indicate bootstrap values equal to or greater than 90.

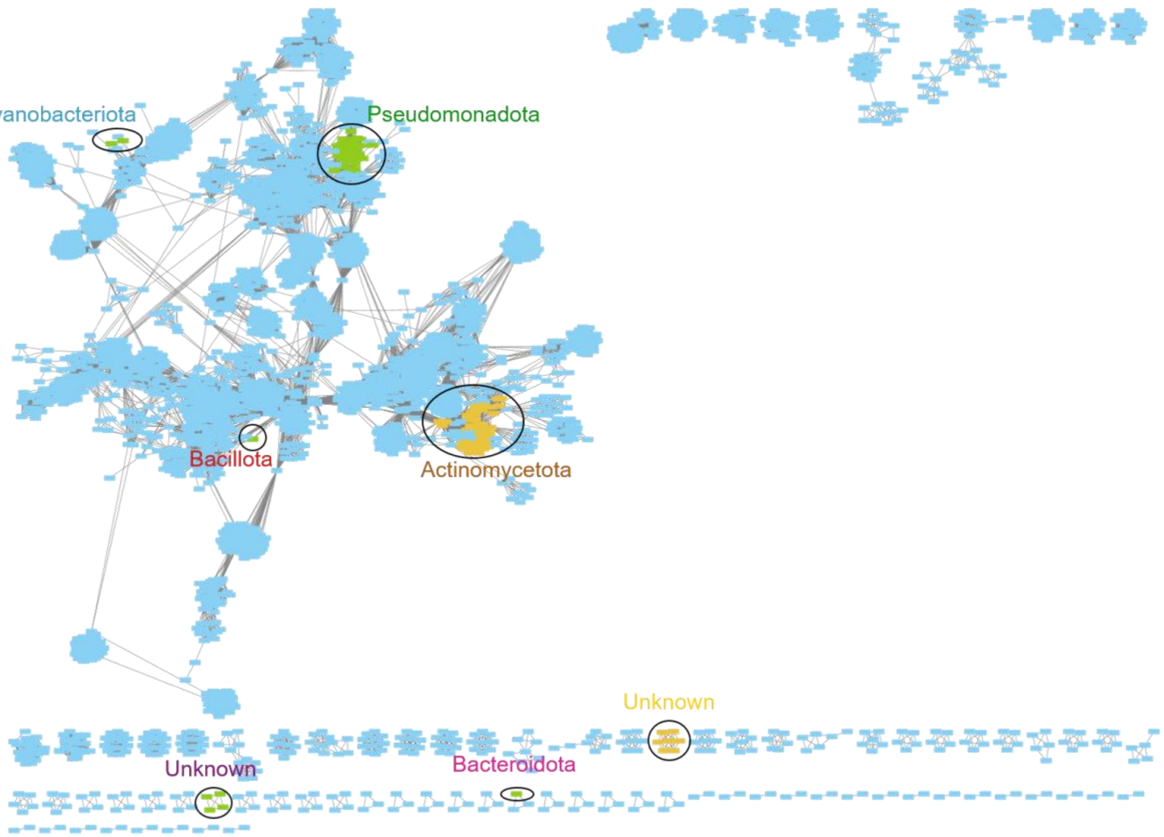

Supplementary Figure 9. Network-based phylogeny of Z-DNA phages. PurZ-containing phages are shown in green, PurZ0-containing phages in yellow and other phages in blue. When possible, the host phylum is indicated.

Primer name	Sequence
X1888	GGGTTAATTAATGTTCGCTAACCCGAACCAGC
X1889	GGGTCTATGCGGCCGCTTACCGGCCGGTACTCCCGAAAC
X1890	GGGTTAATTAATGACCCCCTACCTGTACTTCG
X1891	GGGTCTATGCGGCCGCTTAAAGTTCAAACGGTCCACAG
X1892	GGGTTAATTAATGACCAAGACGCTATACCTCGCC
X1893	GGGTCTATGCGGCCGCTTACTTACCGGACGAACC
X3825	GGGCATATGGTCGAATATGTAATCCACCACGCG
X4086	GGGTCTATGCGGCCGCGAGGATGAACCTGTCGTCCATTTC
X3441	GGGTTAATTAAACCCGCAGCGGGAGCGCAAGACC
X3442	GGGTCTATGCGGCCGCTTACTTGGCGAACGCGGGCAGC
X4151	TACCGTTGGCCGTCCAGTTCGCCCTGGACCG
X4152	AACTGGACGGCCAACGGTACTCCCACAGTGG
X4167	TGTTCGAAGCCGCCGAGGCCTGGACGC
X4168	GCCTCGGCGGCTTCGAACACCACCGCCTTCAC
X4031	AGCTGGCCGACCTGGCGGTCCTGGACCTGTG
X4032	ACCGCCAGGTCGGCCAGCTGTAGGGTACCGTC
X4371	GGGCATATGACCCCCTACCTGGCCTTCGCACGCCCGGTGG
X4149	GGGCATATGACCCCCTACCTGTACTTCGCACGCCCGGTGGCGCAGGCACCGGCCTCG
X4150	GGGCATATGACCCCCTACCTGTACTTCGCACGCCCGGTGCGTCAGGCACCGGCCTCG

Supplementary Table 1. List of primers

Organism	Protein name	PDB ID	TM-score	RMSD
Rattus norvegicus	DNPH1	4KXL	0.78	2.28
Homo sapiens	DNPH1	4P5E	0.76	2.74
Streptomyces rimofaciens	MilB	4JEM	0.76	2.87
Bacillus psychrosaccharolyticus		6EVS	0.76	2.71
Desulfotalea psychrophila LSv54		7O62	0.75	2.49
Enterococcus faecalis V583		7M5H	0.67	2.55
Lactobacillus leichmannii	Ntd	4HX9	0.71	2.85

Supplementary Table 2. Structural homologs of Arthrobacter phage Wayne Dut-like N-ter domain from PDB database. Tm-score and RMSD were calculated using Foldseek.

Ecosystem	PurZ (%)	PurZ0 (%)
Soil	2	91.8
Freshwater	10.2	8.2
Marine	10.2	0
Wastewater	14.2	0
Animal-associated	14.2	0
Hospital	8.2	0
Other	16.3	0
Unknown	24.7	0

Supplementary Table 3. Environmental distribution of the 151 Z-genomes. Z-genomes have been divided into PurZ (n=49) and PurZ0-encoding phages (n=98).
